# Volume Integral Equation Method Solution for Spheroidal Inclusion Problem

**DOI:** 10.3390/ma14226996

**Published:** 2021-11-18

**Authors:** Jungki Lee, Mingu Han

**Affiliations:** Department of Mechanical and Design Engineering, Hongik University, Sejong City 30016, Korea; pooh9265@naver.com

**Keywords:** volume integral equation method (VIEM), isotropic/anisotropic inclusion problems, boundary element method (BEM), standard finite element method (FEM)

## Abstract

In this paper, the volume integral equation method (VIEM) is introduced for the numerical analysis of an infinite isotropic solid containing a variety of single isotropic/anisotropic spheroidal inclusions. In order to introduce the VIEM as a versatile numerical method for the three-dimensional elastostatic inclusion problem, VIEM results are first presented for a range of single isotropic/orthotropic spherical, prolate and oblate spheroidal inclusions in an infinite isotropic matrix under uniform remote tensile loading. We next considered single isotropic/orthotropic spherical, prolate and oblate spheroidal inclusions in an infinite isotropic matrix under remote shear loading. The authors hope that the results using the VIEM cited in this paper will be established as reference values for verifying the results of similar research using other analytical and numerical methods.

## 1. Introduction

The matrix and fibers in composites are usually made of isotropic material. However, in order to have higher strength and stiffness for commercial use, especially in the aerospace and automobile sectors, some constituents of metal matrix composites can be anisotropic. Since anisotropic materials are able to enhance mechanical properties toward orientation, certain mechanical properties (e.g., tensile strength) of anisotropic materials thus depend on orientation. As an example, in titanium-silicon carbide (Ti-SiC) composites, the matrix is nearly isotropic, but the SiC fiber has strong anisotropy and a multilayered structure including an interphase and a core.

A number of analytical techniques for solving inclusion problems are available when the inclusions are simple two-dimensional shapes (cylindrical and elliptical) or simple three-dimensional shapes (spherical and ellipsoidal) and when they are well-separated [1,2,3,4,5]. In particular, Eshelby developed a simple and elegant method for solving the inclusion problem in isotropic solids in 1957 [1]. Eshelby first pointed out that the resulting elastic field can be found with the help of a sequence of imaginary cutting, straining and welding operations [1]. Eshelby also found that the strain and stress field inside the ellipsoidal inclusion is uniform and has a closed-form solution, regardless of the material properties and initial eigenstrain [1]. Eshelby’s findings significantly influenced the mechanics of composites.

In the micromechanical analysis of composite materials, it is often assumed that the inclusions are periodically distributed in the matrix. Then, the unit-cell model with periodic boundary conditions is used to evaluate the overall, microstructure-insensitive, material properties of the composite. However, in real composites, the distribution of the inclusions is not periodic. Thus, the unit-cell model may not provide accurate estimates of the failure and damage mechanisms in composites [6,7,8].

Therefore, stress analysis of heterogeneous solids often requires the use of numerical approaches based on the standard finite element or boundary element formulations. However, both methods present difficulties in dealing with problems involving infinite media or multiple anisotropic inclusions. In response to this concern, it has been demonstrated that the volume integral formulation can overcome both of these limitations in heterogeneous problems involving infinite media [9,10,11].

In comparison to the boundary element method (BEM), the volume integral equation method (VIEM) does not require the use of the Green’s function for anisotropic inclusions and is not sensitive to the geometry of the inclusions. Moreover, as opposed to the standard finite element method (FEM), where it is necessary to discretize the full domain, the multiple inclusions only need to be discretized in the VIEM.

In this paper, three-dimensional elastostatic inclusion problems using the volume integral equation method (VIEM) will be investigated.

In order to introduce the VIEM as a versatile numerical method for the three-dimensional elastostatic inclusion problem, we first examine single isotropic/orthotropic spherical, prolate and oblate spheroidal inclusions in an infinite isotropic matrix subject to uniform remote tensile loading. Two different prolate and oblate spheroidal inclusions with an aspect ratio of 0.5 and 0.75 are considered, respectively. The matrix is assumed to be isotropic. Eight isotropic and five orthotropic inclusions with different characteristics are considered in the numerical calculation. The normalized tensile stress inside the inclusions is investigated in two different directions. Next, we examine single isotropic/orthotropic spherical, prolate and oblate spheroidal inclusions in an infinite isotropic matrix subject to remote shear loading. Two different prolate and oblate spheroidal inclusions with an aspect ratio of 0.5 and 0.75 are considered, respectively. The matrix is assumed to be isotropic. Three isotropic and two orthotropic inclusions with different characteristics are considered in the numerical calculation. The normalized shear stress inside the inclusions is investigated in two different directions.

The authors hope that the present solutions using the parallel volume integral equation method for the single isotropic/orthotropic spherical, prolate and oblate spheroidal inclusions with different material properties under uniform remote tensile loading or remote shear loading will be established as reference values for verifying the results of other analytical and numerical methods.

Since the VIEM is a combination of two powerful general-purpose numerical methods, the standard finite element method (FEM) and the boundary element method (BEM), it is also a highly beneficial tool in the field of numerical analysis and can play a very important role in solving inclusion problems. Subsequently, the purpose of this paper is to introduce the parallel volume integral equation method (PVIEM) as an accessible, versatile and powerful numerical method for solving inclusion problems in the areas of computational mechanics and mechanics of composite materials.

## 2. Governing Equations of Volume Integral Equation Formulation

**Figure 1:**
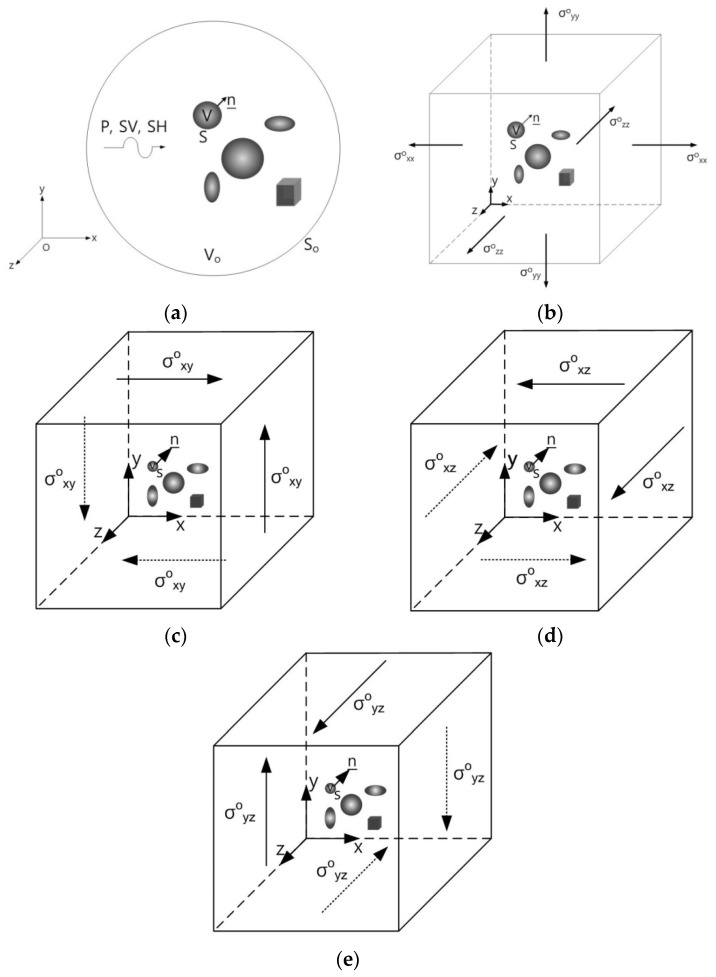
Geometry of the general (**a**) elastodynamic and (**b**) elastostatic problem. (**c**) A remote shear loading, σ^o^_xy_. (**d**) A remote shear loading, σ^o^_xz_. (**e**) A remote shear loading, σ^o^_yz_.

The geometry of the general elastodynamic problem is shown in Figure 1a, where an infinite homogeneous, isotropic and linearly elastic solid containing a number of isotropic or anisotropic inclusions of arbitrary number and shape are subjected to prescribed dynamic loading at infinity.

In Figure 1a, V and S represent the volume and surface of the inclusion respectively, and n is the outward unit normal to S while V_o_ and S_o_ represent the infinite volume and surface, respectively. 

The symbols ρ^(1)^ and c_ijkl_^(1)^ denote the density and the elastic stiffness tensor of the inclusion, while ρ^(^^2)^ and c_ijkl_^(2)^ denote the density and the elastic stiffness tensor of the infinite homogeneous, isotropic and linearly elastic matrix material, respectively. Therefore, c_ijkl_^(2)^ is a constant isotropic tensor, while c_ijkl_^(1)^ can be arbitrary, i.e., the inclusions may, in general, be inhomogeneous and anisotropic. The isotropic or anisotropic inclusions are assumed to be perfectly bonded to the matrix.

Mal and Knopoff [12] showed that the elastodynamic displacement, u_m_(**x**), in the composite satisfies the volume integral equation:

(1) $$u_{m}(x)=u_{m}^{o}(x)+\int_{V}\left[\delta \rho \omega^{2}g_{i}^{m}(\xi ,x)u_{i}(\xi \right)-\delta c_{ijkl}g_{i,j}^{m}(\xi ,x)u_{k,l}(\xi )]d\xi$$

where the integral is over the domain V occupied by the isotropic or anisotropic inclusions, δρ = ρ^(1)^ − ρ^(2)^ and δc_ijkl_ = c_ijkl_^(1)^ − c_ijkl_^(2)^, and g_i_^m^(**ξ**,**x**) is the elastodynamic Green’s function for the infinite homogeneous, isotropic and linearly elastic matrix material.

In Equation (1), u_m_^o^(**x**, **ω**)e^−iωt^ represents the *m*th component of the displacement vector due to the incident field at **x** in the absence of the inclusions, while u_m_(**x**, **ω**)e^−iωt^ denotes the same quantity in the presence of the isotropic or anisotropic inclusions, where **ω** is the circular frequency of the waves. In what follows, the explicit dependence on the circular frequency, and the common time factor, e^−iωt^, for all field quantities will be suppressed.

The geometry of the general elastostatic problem is shown in Figure 1b–e. It has been shown by Lee and Mal [9] that the corresponding elastostatic displacement, u_m_(**x**), within the composite, fulfills the volume integral equation as:

(2) $$u_{m}(x)=u_{m}^{o}(x)-\int_{V}\delta c_{ijkl}g_{i,j}^{m}(\xi ,x)u_{k,l}(\xi )d\xi$$

where the integral is over the space V occupied by the isotropic or anisotropic inclusions and δc_ijkl_ = c_ijkl_^(1)^ − c_ijkl_^(2)^. The value g_i_^m^(**ξ**,**x**) represents the elastostatic Kelvin’s solution (or Green’s function) for the infinite homogeneous, isotropic and linearly elastic matrix material.

In Equations (1) and (2), the differentiations are with respect to the integration variable, ξ_i_, and the summation convention and comma notation have been utilized. The integrand is non-zero within the isotropic or anisotropic inclusions only, since δc_ijkl_ = 0 outside the inclusions.

If **x** lies inside the inclusions, then Equations (1) and (2) are integro-differential equations for the unknown displacement vector **u**(**x**) within the inclusions. It should be noted that an algorithm was developed by Lee and Mal [9,10] to numerically calculate the unknown displacement vector **u**(**x**) by discretizing the inclusions only using standard finite elements. Once **u**(**x**) within the inclusions is determined, the displacement field outside the inclusions can be obtained from Equations (1) and (2) by evaluating the corresponding integrals respectively, and the stress field within and outside the inclusions can also be readily determined. 

The volume integral equation method (VIEM) was originated from Lee and Mal [10] in 1995. Since 1995, Lee and his co-workers (e.g., [9,10,11,13,14,15,16,17]) have been developing a more engineering-oriented VIEM, while Buryachenko (e.g., [18,19,20]) has been examining a more mathematically oriented VIEM since 2000. Additionally, Dong has conducted research on the volume integral equation method since 2003 [21]. Therefore, the VIEM is broadening its influence on computational fields of study. 

Furthermore, Section 4.3 entitled ‘Volume Integral Equation Method’ of the book “Micromechanics of Heterogeneous Materials” by Buryachenko [18] also explains further mathematical formulation of the elastostatic volume integral equation method. In particular, a general description of the volume integral equation method is presented in Chapter 4 entitled ‘Volume Integral Equation Method (VIEM)’ of the book “Advances in Computers and Information in Engineering Research, Vol. 2” by Michopoulos et al. (eds.) [22]. In addition, complete descriptions of the fundamental numerical technique of Equation (2) can be found in [17] for three-dimensional elastostatic problems.

Although each numerical method has certain advantages, specific disadvantages have led to further discussion and research. For example, in Section 3.1 of Reference [20], Buryachenko points out that the VIEM is quite time-consuming. Moreover, no optimized commercial software exists for its application. 

**Figure 2:**
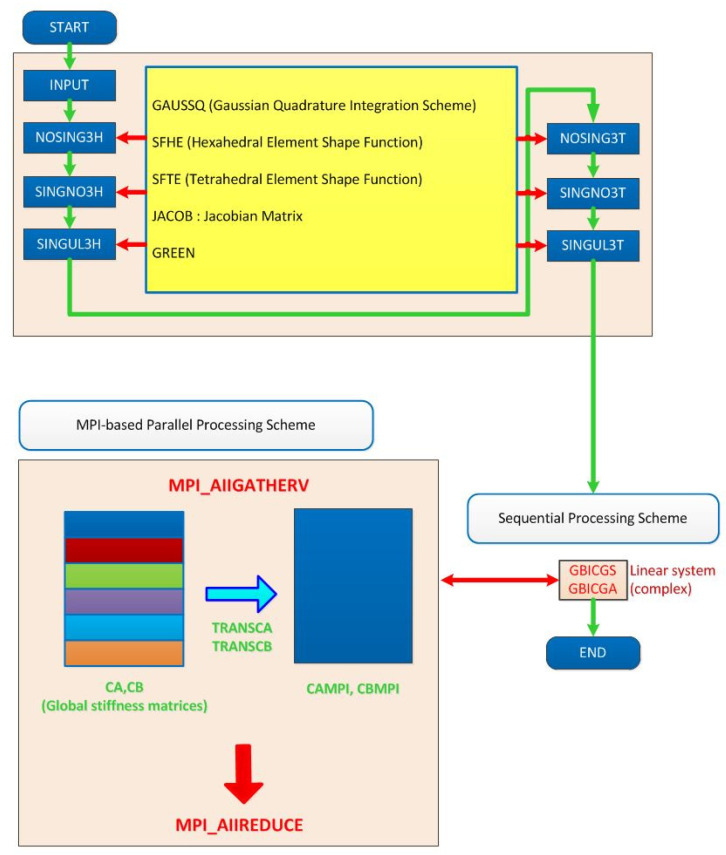
Procedures of ‘pvi3ds01_sm7560xx.f90’ using MPI parallelization.

Firstly, in order to resolve this ‘time-consuming’ problem, we propose the parallel volume integral equation method and implement MPI-based code. Such method allows us not only to solve the large domain but also to speed up computation in the volume integral equation method. The FORTRAN 90 (Version 1.1, IBM, Armonk, NY, USA) source code containing about 9000 lines for the three-dimensional VIEM of the previous paper [17] was parallelized and optimized for this paper, with support from the Korea Institute of Science and Technology Information (KISTI, Daejeon, Korea). Figure 2 shows the procedures of a representative MPI parallelization approach (“pvi3ds01_sm7560xx.f90”) for the sequential three-dimensional VIEM code (“svi3ds01_sm4320xx.f”). As a result, the program execution time has been greatly reduced. Furthermore, we could use more finite elements (31,857 nodes and 7560 elements) in the VIEM model of this paper than those (18,109 nodes and 4320 elements) in the VIEM model of the previous paper [17]. The parallel FORTRAN source code for the three-dimensional VIEM is presently being processed in the KISTI-5. It is referred to as “Nurion”, which is a system consisting of compute nodes, CPU-only nodes, Omni-Path interconnect networks, Burst Buffer high-speed storage, a Luster-based parallel file system and a water-cooling device based on a Rear Door Heat Exchanger (RDHx). The CPU-only nodes consist of 132 Intel Xeon 6148 2.4 GHz processors (named “Skylake”). The total theoretical performance is 25.7 petaflops, which ranked 11th in the world in June 2018 (http://www.top500.org, accessed on 3 May 2021). It should be noted that, in order to investigate three-dimensional stress problems with multiple inclusions, in addition to parallelization and optimization of the sequential three-dimensional VIEM code, a domain decomposition method (DDM) was applied to the parallel three-dimensional VIEM code, with support from the Korea Institute of Science and Technology Information (KISTI). The domain decomposition method allows decomposition of large-sized problem solutions to solutions of several smaller-sized problems [23]. Therefore, the parallel volume integral equation method (PVIEM) using the domain decomposition method enables us to investigate more complicated multiple inclusion problems elastostatically or elastodynamically.

**Figure 3:**
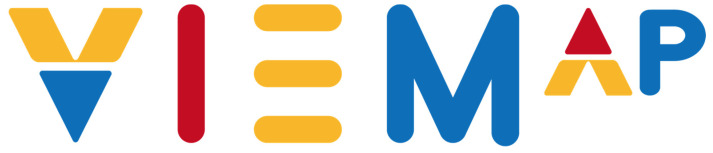
Registered trademark for VIEMAP.

Secondly, in order to resolve the ‘no optimized commercial software’ problem, we plan to develop a semi-commercial VIEM software called the “Volume Integral Equation Method Application Program” (VIEMAP). Table 1 shows the analysis capabilities of VIEMAP including a pre-processor (ViemMesh), a solver (VIEM) and a post-processor (ViemPlot) adapted to solve multiple isotropic/anisotropic inclusion problems in a computationally tractable manner. Figure 3 shows the registered trademark for the VIEMAP. The authors aim to help both university students and researchers create VIEM models using the VIEMAP more easily than using the standard finite element method (FEM), as well as solve multiple isotropic/anisotropic inclusion problems in an unbounded isotropic medium more accurately and conveniently than the boundary element method (BEM).

## 3. Three-Dimensional Elastostatic Problems Using the VIEM

**Figure 4:**
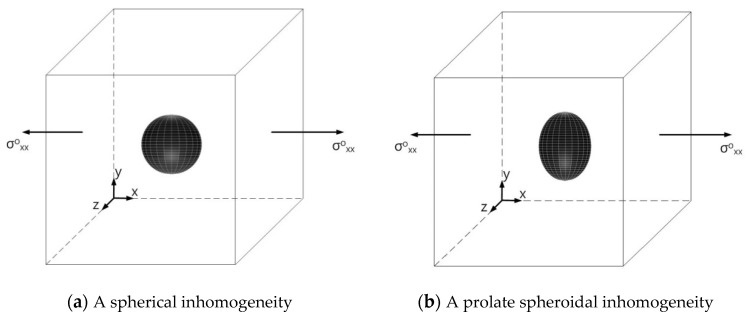

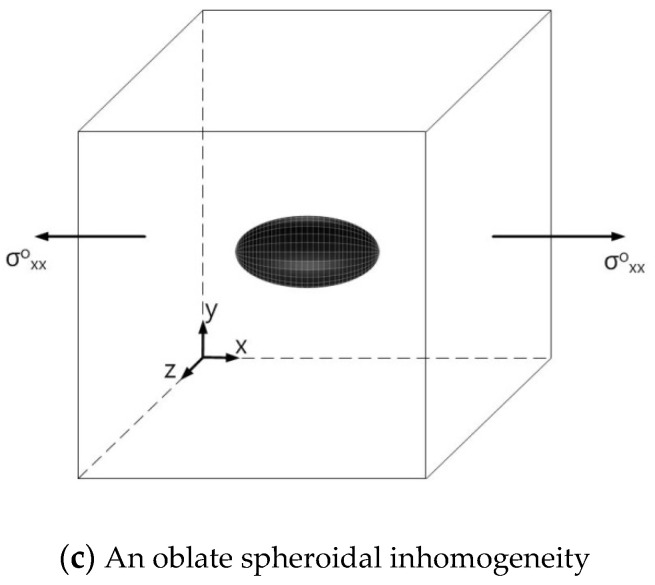
(**a**) Spherical, (**b**) prolate spheroidal and (**c**) oblate spheroidal inclusions under uniform remote tensile loading (σ^o^_xx_).

**Figure 5:**
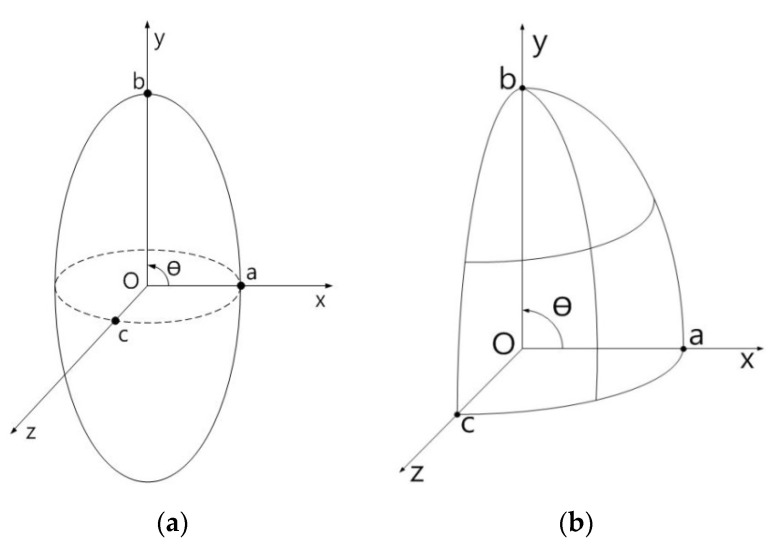
The orientation of spherical, prolate spheroidal and oblate spheroidal inclusions. (**a**) Spheroidal coordinate system. (**b**) Cartesian coordinate system.

In this section, we first examine single isotropic/orthotropic spherical, prolate and oblate spheroidal inclusions in an infinite isotropic matrix subject to uniform remote tensile loading, σ^o^_xx_, as shown in Figure 4 (also see Figure 1b and Figure 5). The remote applied load can be arbitrarily chosen and was assumed to be σ^o^_xx_ = 143.10 GPa for convenience purposes only. Two different prolate spheroidal inclusions were considered: (a) a/b = c/b = 0.5 and (b) a/b = c/b = 0.75 (see Figure 5). Additionally, two different oblate spheroidal inclusions were considered: (a) b/a = c/a = 0.5 and (b) b/a = c/a = 0.75 (see Figure 5).

The elastic constants for the isotropic matrix and the isotropic inclusions are listed in Table 2. The elastic constants for the isotropic matrix and the orthotropic inclusions are listed in Table 3.

**Figure 6:**
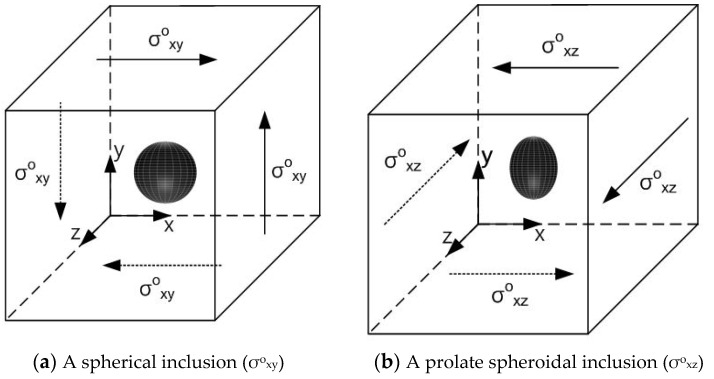

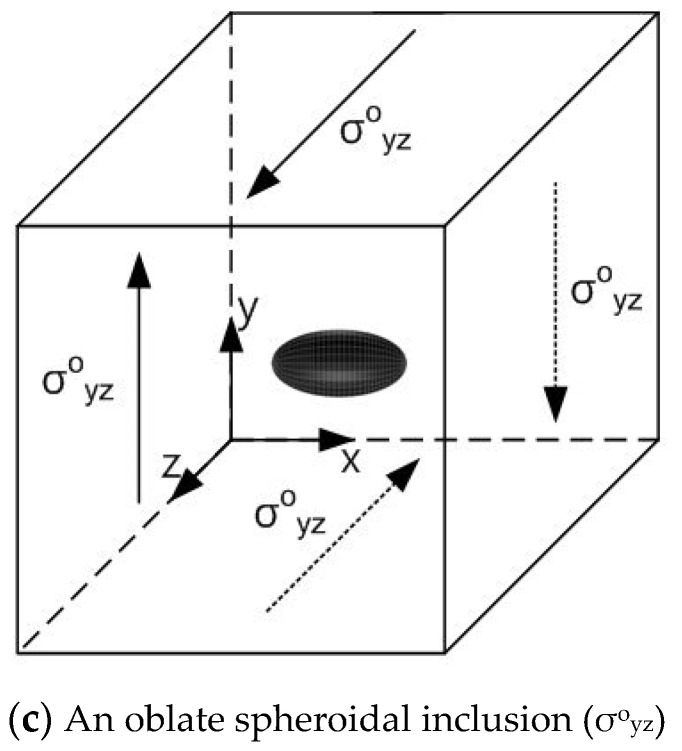
(**a**) Spherical, (**b**) prolate spheroidal and (**c**) oblate spheroidal inclusions under remote shear loading.

We next examine single isotropic/orthotropic spherical, prolate and oblate spheroidal inclusions in an infinite isotropic matrix subject to remote shear loading, σ^o^_x__y_, σ^o^_x__z_ or σ^o^_yz_, as shown in Figure 6 (also see Figure 1c–e and Figure 5) [24]. The remote applied load can be arbitrarily chosen and was assumed to be σ^o^_x__y_ = σ^o^_x__z_ = σ^o^_yz_ = 75.76 GPa for convenience purposes only. We considered the same geometry of the single spherical, prolate (with an aspect ratio of 0.5 and 0.75) and oblate (with an aspect ratio of 0.5 and 0.75) spheroidal inclusions in an infinite isotropic matrix under remote shear loading (σ^o^_x__y_, σ^o^_x__z_ and σ^o^_yz_).

Three different material properties (Iso_01, Iso_05 and Iso_06) in Table 2 were used in the numerical calculation. The elastic constants for the isotropic matrix and the orthotropic inclusions are listed in Table 4. Table 5 shows various characteristics of the material properties used in the numerical calculation. In order to demonstrate the capability of the volume integral equation method for the three-dimensional anisotropic inclusion problem, three independent elastic constants, c_44_ (shear modulus in the yz plane), c_55_ (shear modulus in the xz plane) and c_66_ (shear modulus in the xy plane), were assumed to be different from each other [25].

### 3.1. Single Spherical Inclusion Problems under Uniform Remote Tensile Loading 

#### 3.1.1. VIEM Formulation Applied to Isotropic Inclusion Problems 

The displacements in the volume integral Equation (2) for isotropic spherical, prolate and oblate spheroidal inclusions can be expressed in the form:

(3) $$\begin{array}{ll} u_{1}(x)= & u_{1}^{o}(x)-\int_{V}\{\delta (\lambda +2\mu )g_{1,1}^{1}u_{1,1}+\delta \lambda (g_{1,1}^{1}u_{2,2}+g_{2,2}^{1}u_{1,1})+\delta \lambda (g_{1,1}^{1}u_{3,3}+g_{3,3}^{1}u_{1,1})\\ & +\delta (\lambda +2\mu )g_{2,2}^{1}u_{2,2}+\delta \lambda (g_{2,2}^{1}u_{3,3}+g_{3,3}^{1}u_{2,2})+\delta (\lambda +2\mu )g_{3,3}^{1}u_{3,3}\\ & +\delta \mu \left[g_{2,3}^{1}\left(u_{2,3}+u_{3,2}\right)+g_{3,2}^{1}\left(u_{2,3}+u_{3,2}\right)\right]\\ & +\delta \mu \left[g_{1,3}^{1}\left(u_{1,3}+u_{3,1}\right)+g_{3,1}^{1}\left(u_{1,3}+u_{3,1}\right)\right]\\ & +\delta \mu \left[g_{1,2}^{1}\left(u_{1,2}+u_{2,1}\right)+g_{2,1}^{1}\left(u_{1,2}+u_{2,1}\right)\right]\}d\xi_{1}d\xi_{2}d\xi_{3} \end{array}$$

(4) $$\begin{array}{ll} u_{2}(x)= & u_{2}^{o}(x)-\int_{V}\{\delta (\lambda +2\mu )g_{1,1}^{2}u_{1,1}+\delta \lambda (g_{1,1}^{2}u_{2,2}+g_{2,2}^{2}u_{1,1})+\delta \lambda (g_{1,1}^{2}u_{3,3}+g_{3,3}^{2}u_{1,1})\\ & +\delta (\lambda +2\mu )g_{2,2}^{2}u_{2,2}+\delta \lambda (g_{2,2}^{2}u_{3,3}+g_{3,3}^{2}u_{2,2})+\delta (\lambda +2\mu )g_{3,3}^{2}u_{3,3}\\ & +\delta \mu \left[g_{2,3}^{2}\left(u_{2,3}+u_{3,2}\right)+g_{3,2}^{2}\left(u_{2,3}+u_{3,2}\right)\right]\\ & +\delta \mu \left[g_{1,3}^{2}\left(u_{1,3}+u_{3,1}\right)+g_{3,1}^{2}\left(u_{1,3}+u_{3,1}\right)\right]\\ & +\delta \mu \left[g_{1,2}^{2}\left(u_{1,2}+u_{2,1}\right)+g_{2,1}^{2}\left(u_{1,2}+u_{2,1}\right)\right]\}d\xi_{1}d\xi_{2}d\xi_{3} \end{array}$$

(5) $$\begin{array}{ll} u_{3}(x)= & u_{3}^{o}(x)-\int_{V}\{\delta (\lambda +2\mu )g_{1,1}^{3}u_{1,1}+\delta \lambda (g_{1,1}^{3}u_{2,2}+g_{2,2}^{3}u_{1,1})+\delta \lambda (g_{1,1}^{3}u_{3,3}+g_{3,3}^{3}u_{1,1})\\ & +\delta (\lambda +2\mu )g_{2,2}^{3}u_{2,2}+\delta \lambda (g_{2,2}^{3}u_{3,3}+g_{3,3}^{3}u_{2,2})+\delta (\lambda +2\mu )g_{3,3}^{3}u_{3,3}\\ & +\delta \mu \left[g_{2,3}^{3}\left(u_{2,3}+u_{3,2}\right)+g_{3,2}^{3}\left(u_{2,3}+u_{3,2}\right)\right]\\ & +\delta \mu \left[g_{1,3}^{3}\left(u_{1,3}+u_{3,1}\right)+g_{3,1}^{3}\left(u_{1,3}+u_{3,1}\right)\right]\\ & +\delta \mu \left[g_{1,2}^{3}\left(u_{1,2}+u_{2,1}\right)+g_{2,1}^{3}\left(u_{1,2}+u_{2,1}\right)\right]\}d\xi_{1}d\xi_{2}d\xi_{3} \end{array}$$

where u_1_(x), u_2_(x) and u_3_(x) are the three-dimensional displacements, δc_αβ_ = c_αβ_(1) − c_αβ_(2) (α, β = 1, 6), where c_αβ_(1) represents the elastic stiffness constants of the isotropic inclusions, while c_αβ_(2) denotes those for the isotropic matrix material: δc_11_ = δc_22_ = δc_33_ = (λ_1_ + 2μ_1_) − (λ_2_ + 2μ_2_), δc_12_ = δc_13_ = δc_23_ = λ_1_ − λ_2_ and δc_44_ = δc_55_ = δc_66_ = μ_1_ − μ_2_. 

In Equations (3)–(5), g_i_^m^(**ξ**,**x**) is the Green’s function for the infinite isotropic matrix material and is stated by Banerjee [26] and Pao and Varatharajulu [27] as:

(6) $$g_{1}^{1}=\frac{1}{16\pi \left(1-\nu \right)\mu r}[\frac{{\left(x_{1}-\xi_{1}\right)}^{2}}{r^{2}}+\left(3-4\nu \right)]\;g_{2}^{1}=g_{1}^{2}=\frac{1}{16\pi \left(1-\nu \right)\mu r}[\frac{\left(x_{1}-\xi_{1})(x_{2}-\xi_{2}\right)}{r^{2}}]\;g_{3}^{1}=g_{1}^{3}=\frac{1}{16\pi \left(1-\nu \right)\mu r}[\frac{\left(x_{1}-\xi_{1})(x_{3}-\xi_{3}\right)}{r^{2}}]\;g_{2}^{2}=\frac{1}{16\pi \left(1-\nu \right)\mu r}[\frac{{\left(x_{2}-\xi_{2}\right)}^{2}}{r^{2}}+\left(3-4\nu \right)]\;g_{3}^{2}=g_{2}^{3}=\frac{1}{16\pi \left(1-\nu \right)\mu r}[\frac{\left(x_{2}-\xi_{2})(x_{3}-\xi_{3}\right)}{r^{2}}]\;g_{3}^{3}=\frac{1}{16\pi \left(1-\nu \right)\mu r}[\frac{{\left(x_{3}-\xi_{3}\right)}^{2}}{r^{2}}+\left(3-4\nu \right)]$$

where r = |x − ξ| = $\sqrt{{\left(x_{1}^{}-\xi_{1}^{}\right)}_{}^{2}+{\left(x_{2}^{}-\xi_{2}^{}\right)}_{}^{2}+{\left(x_{3}^{}-\xi_{3}^{}\right)}_{}^{2}}$, ν is Poisson’s ratio and μ is the shear modulus for the infinite isotropic matrix material.

#### 3.1.2. VIEM Formulation Applied to Orthotropic Inclusion Problems

Let the coordinate axes x_1_(x), x_2_(y) and x_3_(z) be taken parallel to the symmetry axes of the orthotropic material, and c_11_, c_12_, c_13_, c_22_, c_23_, c_33_, c_44_, c_55_ and c_66_ denote the elastic constants. The displacements in Equation (2) for orthotropic spherical, prolate and oblate spheroidal inclusions can be expressed in the form:

(7) $$\begin{array}{ll} u_{1}(x)= & u_{1}^{o}(x)-\int_{V}\{\delta c_{11}g_{1,1}^{1}u_{1,1}+\delta c_{12}(g_{1,1}^{1}u_{2,2}+g_{2,2}^{1}u_{1,1})+\delta c_{13}(g_{1,1}^{1}u_{3,3}+g_{3,3}^{1}u_{1,1})\\ & +\delta c_{22}g_{2,2}^{1}u_{2,2}+\delta c_{23}(g_{2,2}^{1}u_{3,3}+g_{3,3}^{1}u_{2,2})+\delta c_{33}g_{3,3}^{1}u_{3,3}\\ & +\delta c_{44}\left[g_{2,3}^{1}\left(u_{2,3}+u_{3,2}\right)+g_{3,2}^{1}\left(u_{2,3}+u_{3,2}\right)\right]\\ & +\delta c_{55}\left[g_{1,3}^{1}\left(u_{1,3}+u_{3,1}\right)+g_{3,1}^{1}\left(u_{1,3}+u_{3,1}\right)\right]\\ & +\delta c_{66}\left[g_{1,2}^{1}\left(u_{1,2}+u_{2,1}\right)+g_{2,1}^{1}\left(u_{1,2}+u_{2,1}\right)\right]\}d\xi_{1}d\xi_{2}d\xi_{3} \end{array}$$

(8) $$\begin{array}{ll} u_{2}(x)= & u_{2}^{o}(x)-\int_{V}\{\delta c_{11}g_{1,1}^{2}u_{1,1}+\delta c_{12}(g_{1,1}^{2}u_{2,2}+g_{2,2}^{2}u_{1,1})+\delta c_{13}(g_{1,1}^{2}u_{3,3}+g_{3,3}^{2}u_{1,1})\\ & +\delta c_{22}g_{2,2}^{2}u_{2,2}+\delta c_{23}(g_{2,2}^{2}u_{3,3}+g_{3,3}^{2}u_{2,2})+\delta c_{33}g_{3,3}^{2}u_{3,3}\\ & +\delta c_{44}\left[g_{2,3}^{2}\left(u_{2,3}+u_{3,2}\right)+g_{3,2}^{2}\left(u_{2,3}+u_{3,2}\right)\right]\\ & +\delta c_{55}\left[g_{1,3}^{2}\left(u_{1,3}+u_{3,1}\right)+g_{3,1}^{2}\left(u_{1,3}+u_{3,1}\right)\right]\\ & +\delta c_{66}\left[g_{1,2}^{2}\left(u_{1,2}+u_{2,1}\right)+g_{2,1}^{2}\left(u_{1,2}+u_{2,1}\right)\right]\}d\xi_{1}d\xi_{2}d\xi_{3} \end{array}$$

(9) $$\begin{array}{ll} u_{3}(x)= & u_{3}^{o}(x)-\int_{V}\{\delta c_{11}g_{1,1}^{3}u_{1,1}+\delta c_{12}(g_{1,1}^{3}u_{2,2}+g_{2,2}^{3}u_{1,1})+\delta c_{13}(g_{1,1}^{3}u_{3,3}+g_{3,3}^{3}u_{1,1})\\ & +\delta c_{22}g_{2,2}^{3}u_{2,2}+\delta c_{23}(g_{2,2}^{3}u_{3,3}+g_{3,3}^{3}u_{2,2})+\delta c_{33}g_{3,3}^{3}u_{3,3}\\ & +\delta c_{44}\left[g_{2,3}^{3}\left(u_{2,3}+u_{3,2}\right)+g_{3,2}^{3}\left(u_{2,3}+u_{3,2}\right)\right]\\ & +\delta c_{55}\left[g_{1,3}^{3}\left(u_{1,3}+u_{3,1}\right)+g_{3,1}^{3}\left(u_{1,3}+u_{3,1}\right)\right]\\ & +\delta c_{66}\left[g_{1,2}^{3}\left(u_{1,2}+u_{2,1}\right)+g_{2,1}^{3}\left(u_{1,2}+u_{2,1}\right)\right]\}d\xi_{1}d\xi_{2}d\xi_{3} \end{array}$$

where u_1_(x), u_2_(x) and u_3_(x) are the three-dimensional displacements, δc_αβ_ = c_αβ_(1) − c_αβ_(2) (α, β = 1, 6), where = c_αβ_(1) represents the elastic stiffness constants of the orthotropic inclusions, while c_αβ_(2) denotes those for the isotropic matrix material: δc_11_ = c_11_ − (λ_2_ + 2μ_2_), δc_22_ = c_22_ − (λ_2_ + 2μ_2_), δc_33_ = c_33_ − (λ_2_ + 2μ_2_), δc_12_ = c_12_ − λ_2_, δc_13_ = c_13_ − λ_2_, δc_23_ = c_23_ − λ_2_ and δc_44_ = c_44_ − μ_2_, δc_55_ = c_55_ − μ_2_, δc_66_ = c_66_ − μ_2_.

In Equations (7)–(9), g_i_^m^(**ξ**,**x**) is the Green’s function for the infinite isotropic matrix material and is stated in Equation (6). Thus, the VIEM does not require the use of the Green’s function for the orthotropic material of the inclusion. In general, Green’s function for an anisotropic material is much more complex than that of an isotropic material [28]. Furthermore, a closed form solution of the generalized Green’s function for an anisotropic material is not available in the literature. 

In contrast, in the BEM, Green’s functions for both the isotropic matrix and the anisotropic inclusions must be specified in the formulation. In particular, special emphasis is placed on the fact that Green’s function for the anisotropic material of the inclusions is not required in the VIEM.

#### 3.1.3. Numerical Formulations in the VIEM

The integrands in Equations (3)–(8) contain singularities with different orders due to the singular characteristics of the Green’s function at x = ξ (i.e., r = 0). Thus, evaluation of the singular integrals requires special attention. In general, g_i_^m^(**ξ**,**x**) behaves as 1/r, while its derivatives behave as 1/r^2^ as r → 0. It should be noted that only g_i_^m^(**ξ**,**x**) for the isotropic matrix and its derivatives are required in the VIEM. Furthermore, in the BEM, the Green’s function for anisotropic inclusions and their derivatives must also be specified. As a result, this may be a critical drawback to the BEM when solving multiple anisotropic inclusion problems. 

In contrast to the BEM, the singularities in the VIEM are integrable (weak). Thus, we have decided to utilize the direct integration scheme stated by Li et al. [29]. Finally, after suitable adjustments, we have succeeded in addressing these weak singular integrands in the volume integral equation formulations.

A comprehensive elaboration for the accurate evaluation of singular integrals using the tetrahedron polar co-ordinates shown in [29] was presented in [17].

#### 3.1.4. A Single Isotropic Spherical Inclusion

**Figure 7:**
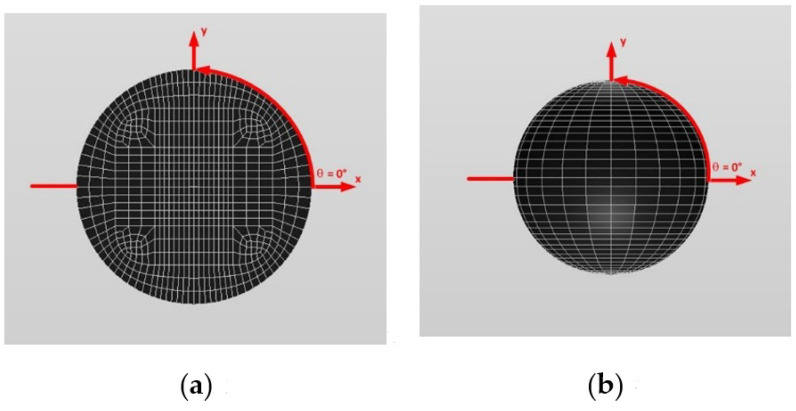
A typical discretized spherical model in the volume integral equation method (VIEM). (**a**) An inside view of a spherical model. (**b**) A spherical model.

**Figure 8:**
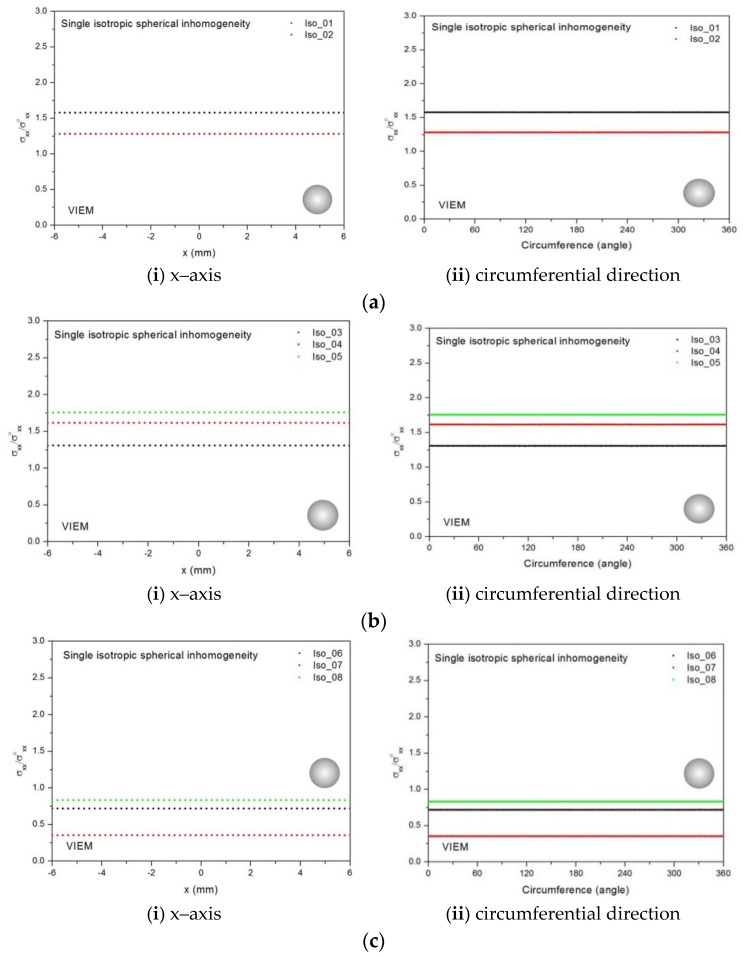
VIEM results for the normalized tensile stress component (σ_xx_/σ^o^_xx_) along (**i**) the x–axis inside and (**ii**) the circumferential direction of the isotropic spherical inclusions with a radius of 6 mm under uniform remote tensile loading. (**a**) Iso_01 and Iso_02. (**b**) Iso_03, Iso_04 and Iso_05. (**c**) Iso_06, Iso_07 and Iso_08.

In order to examine the accuracy of the numerical results using the VIEM, the numerical results using the VIEM for a single isotropic spherical inclusion were first compared to the analytical solutions [21,30]. We considered a single isotropic spherical inclusion with a radius of 6 mm in an infinite isotropic matrix subject to uniform remote tensile loading, σ_xx_^o^, as shown in Figure 4a. It should be noted that the length of the radius can be arbitrarily chosen. In Figure 7, standard 20-node quadratic hexahedral elements were used in the discretization [31]. The number of hexahedral elements, 7560, was determined based on a convergence test. For the seven different material properties (Iso_2, Iso_03, Iso_04, Iso_05, Iso_06, Iso_07 and Iso_08) in Table 2, a comparison was made between the numerical results using the volume integral equation method (VIEM) and the analytical solutions. As shown in Table 5, there was no restriction to Poisson’s ratio in the inclusions and matrices of Iso_02 and Iso_06. However, Poison’s ratio was 1/3 in both the inclusion and matrix of Iso_03, Iso_04, Iso_05, Iso_07 and Iso_08. Furthermore, for Iso_02, Iso_03, Iso_04 and Iso_05, Young’s modulus (E) in the isotropic inclusion was greater than that in the isotropic matrix. For Iso_06, Iso_07 and Iso_08, Young’s modulus (E) in the isotropic matrix was greater than that in the isotropic inclusion. Thus, seven material properties representing a diversity of materials were chosen. Excellent agreement was found between the analytical and numerical solutions using the VIEM for the seven different materials considered. It should be noted that the VIEM results represent average values of the normalized stresses in all the nodes of the VIEM model in Figure 7. It should also be noted that the normalized tensile stress (σ_xx_/σ^o^_xx_) inside the isotropic spherical inclusions was found to be constant [1,30]. Table 6, Table 7 and Table 8 show that the percentage differences for the two sets of results are less than 0.1% in seven cases. Figure 8 shows numerical solution by the volume integral equation method for the normalized tensile stress (σ_xx_/σ^o^_xx_) along (i) the x–axis inside (−6 mm ≤ x ≤ 6 mm) and (ii) the circumferential direction (0° ≤ θ (see Figure 7) ≤ 360°) of the isotropic spherical inclusions with a radius of 6 mm under uniform remote tensile loading. 

In most references, the numerical results for this problem were obtained in one direction. Thus, in order to show the VIEM results more thoroughly, the normalized tensile stress (σ_xx_/σ^o^_xx_) using the VIEM was presented along (i) the x–axis inside (−6 mm ≤ x ≤ 6 mm) and (ii) the circumferential direction (0° ≤ θ (see Figure 7) ≤ 360°) of the isotropic spherical inclusions. It was determined in Figure 8 that the normalized tensile stress (σ_xx_/σ^o^_xx_) inside the isotropic spherical inclusions is constant in all directions considered.

#### 3.1.5. A Single Orthotropic Spherical Inclusion

In order to show the advantages of the volume integral equation method (VIEM), we consider a single orthotropic spherical inclusion with a radius of 6 mm in an infinite isotropic matrix subject to uniform remote tensile loading, σ^o^_xx_, as shown in Figure 4a. It should be noted that the length of the radius can be arbitrarily chosen. In Figure 7, standard 20-node quadratic hexahedral elements were used in the discretization [31]. The number of hexahedral elements was 7560, determined based on a convergence test. For this problem, in comparison to the boundary element method (BEM), since the VIEM is not sensitive to the anisotropy of the inclusions, it does not require use of the Green’s function for the anisotropic inclusions. Moreover, as opposed to the standard FEM, where it is necessary to discretize the full domain, the orthotropic inclusion only needs to be discretized in the VIEM.

**Figure 9:**
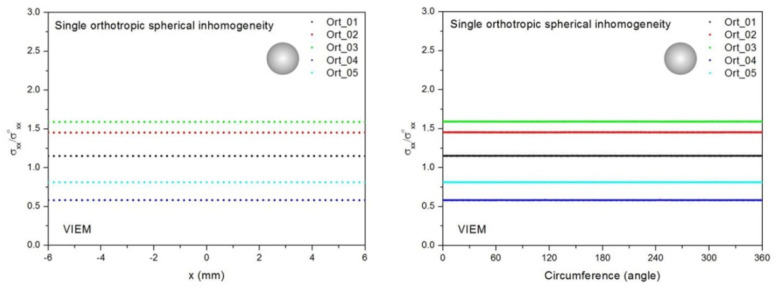
VIEM results for the normalized tensile stress component (σ_xx_/σ^o^_xx_) along (**left**) the x–axis inside and (**right**) the circumferential direction of the orthotropic spherical inclusions (Ort_01, Ort_02, Ort_03, Ort_04 and Ort_05) with a radius of 6 mm under uniform remote tensile loading.

Five different material properties (Ort_1, Ort_02, Ort_03, Ort_04 and Ort_05) in Table 5 were used in the numerical calculation. As shown in Table 5, it was assumed that c_11_ > c_22_ = c_33_ for five orthotropic inclusions. Additionally, c_11_ of the inclusion in Ort_03 > c_11_ of the inclusion in Ort_02 > c_11_ of the inclusion in Ort_01. Furthermore, c_11_ of the inclusion in Ort_04 < c_11_ of the inclusion in Ort_05 < c_11_ of the inclusion in Ort_01. Thus, five material properties representing a diversity of materials were chosen. It should be noted that the VIEM results represent average values of the normalized stresses in all the nodes of the VIEM model in Figure 7. Moreover, the normalized tensile stress (σ_xx_/σ^o^_xx_) inside the orthotropic spherical inclusions was found to be constant [1,30]. Table 9 shows the numerical solution by the volume integral equation method for the normalized tensile stress (σ_xx_/σ^o^_xx_) inside the orthotropic spherical inclusions. For the inclusions in Ort_01, Ort_02 and Ort_03, the normalized tensile stress (σ_xx_/σ^o^_xx_) inside the inclusion was greater than 1.0. However, for the inclusions in Ort_04 and Ort_05, the normalized tensile stress (σ_xx_/σ^o^_xx_) inside the inclusion was less than 1.0. Figure 9 shows the numerical solution by the volume integral equation method for the normalized tensile stress (σ_xx_/σ^o^_xx_) along (left) the x–axis inside (−6 mm ≤ x ≤ 6 mm) and (right) the circumferential direction (0° ≤ θ (see Figure 7) ≤ 360°) of the orthotropic spherical inclusions with a radius of 6 mm under uniform remote tensile loading. It was determined in Figure 9 that the normalized tensile stress (σ_xx_/σ^o^_xx_) inside the orthotropic spherical inclusions is constant in all directions considered.

### 3.2. A Single Spheroidal Inclusion Problem under Uniform Remote Tensile Loading

In order to introduce the VIEM as a versatile numerical method, we considered a single isotropic/orthotropic spheroidal inclusion in an infinite isotropic matrix subject to uniform remote tensile loading, σ^o^_xx_, as shown in Figure 4b,c. Figure 5 shows an orientation of the spheroidal inclusion.

#### 3.2.1. A Single Isotropic Prolate Spheroidal Inclusion

Two different prolate spheroidal inclusions are considered: (a) a/b = c/b = 0.5, where b = 6 mm, and (b) a/b = c/b = 0.75, where b = 6 mm (see Figure 5). It should be noted that the length of b (=6 mm) can be arbitrarily chosen.

**Figure 10:**
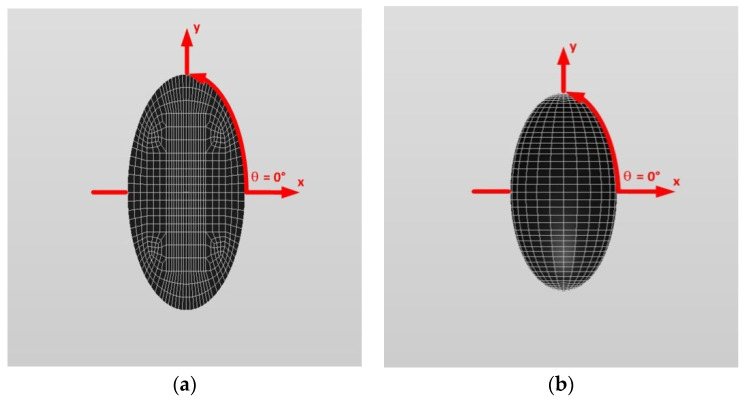
A typical discretized prolate spheroidal model (a/b = c/b = 0.5) in the volume integral equation method (VIEM). (**a**) An inside view of a prolate spheroidal model. (**b**) A prolate spheroidal model.

**Figure 11:**
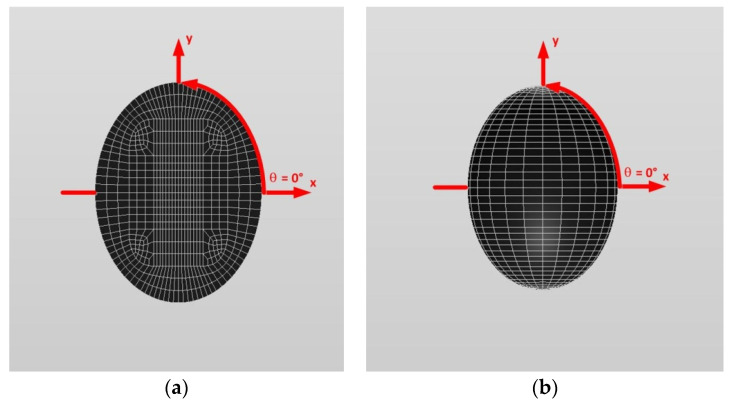
A typical discretized prolate spheroidal model (a/b = c/b = 0.75) in the volume integral equation method (VIEM). (**a**) An inside view of a prolate spheroidal model. (**b**) A prolate spheroidal model.

Figure 10 and Figure 11 show a typical discretized model for the single (a) prolate spheroidal inclusion (a/b = c/b = 0.5 where b = 6 mm) and (b) prolate spheroidal inclusion (a/b = c/b = 0.75 where b = 6 mm) used in the VIEM [31], respectively. A total of 7560 standard 20-node quadratic hexahedral elements were used for the single prolate spheroidal inclusion in Figure 10 and Figure 11. The number of elements, 7560, was determined based on a convergence test. 

Eight different isotropic inclusions (from Iso_01 to Iso_08) in Table 2 were used in the numerical calculation. It should be noted that the VIEM results represent average values of the normalized stresses in all the nodes of the VIEM model in Figure 10 and Figure 11. It should also be noted that the normalized tensile stress (σ_xx_/σ^o^_xx_) inside the isotropic prolate spheroidal inclusions was found to be constant [1,30].

**Figure 12:**
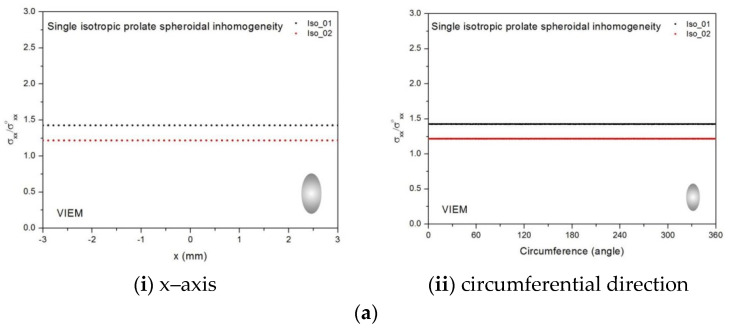

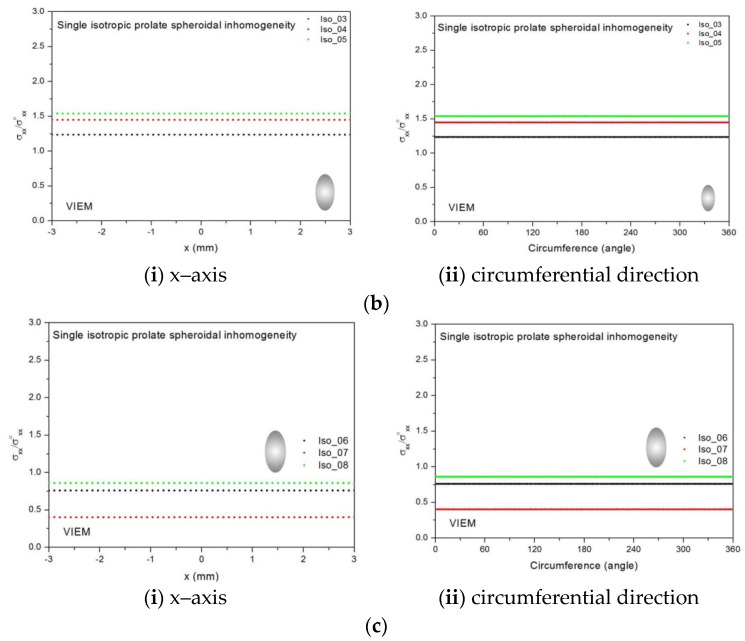
VIEM results for the normalized tensile stress component (σ_xx_/σ^o^_xx_) along (**i**) the x–axis inside and (**ii**) the circumferential direction of the isotropic prolate spheroidal inclusions with a/b = c/b = 0.5 (b = 6 mm) under uniform remote tensile loading. (**a**) Iso_01 and Iso_02. (**b**) Iso_03, Iso_04 and Iso_05. (**c**) Iso_06, Iso_07 and Iso_08.

**Figure 13:**
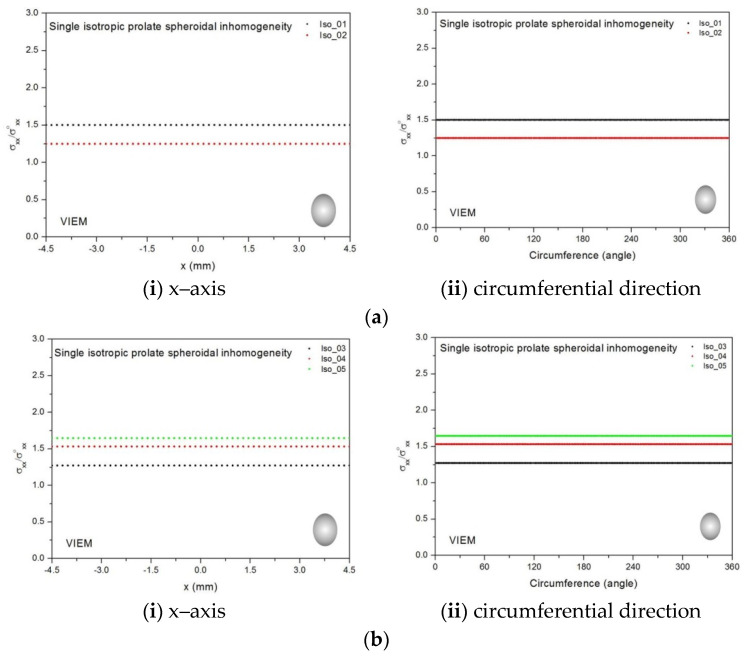

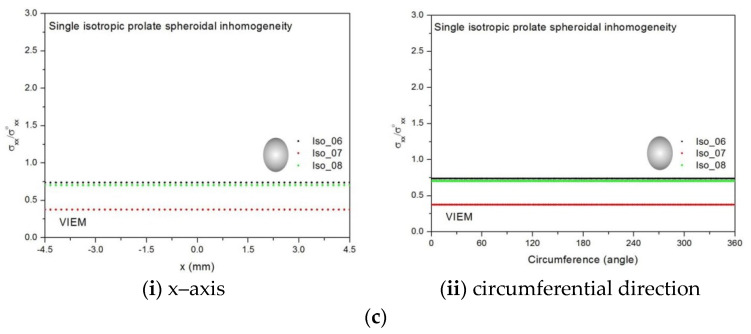
VIEM results for the normalized tensile stress component (σ_xx_/σ^o^_xx_) along (**i**) the x–axis inside and (**ii**) the circumferential direction of the isotropic prolate spheroidal inclusions with a/b = c/b = 0.75 (b = 6 mm) under uniform remote tensile loading. (**a**) Iso_01 and Iso_02. (**b**) Iso_03, Iso_04 and Iso_05. (**c**) Iso_06, Iso_07 and Iso_08.

Table 10, Table 11 and Table 12 show numerical solutions by the volume integral equation method for the normalized tensile stress (σ_xx_/σ^o^_xx_) inside the isotropic prolate spheroidal inclusions. For the inclusions in Iso_01, Iso_02, Iso_03, Iso_04 and Iso_05, the normalized tensile stress (σ_xx_/σ^o^_xx_) inside the inclusion was greater than 1.0. However, for the inclusions in Iso_06, Iso_07 and Iso_08, the normalized tensile stress (σ_xx_/σ^o^_xx_) inside the inclusion was less than 1.0. Figure 12 shows numerical solutions by the volume integral equation method for the normalized tensile stress (σ_xx_/σ^o^_xx_) along (i) the x–axis inside (−3 mm ≤ x ≤ 3 mm) and (ii) the circumferential direction (0° ≤ θ (see Figure 10) ≤ 360°) of the isotropic prolate spheroidal inclusions (a/b = c/b = 0.5 where b = 6 mm) under uniform remote tensile loading. Figure 13 shows numerical solutions by the volume integral equation method for the normalized tensile stress (σ_xx_/σ^o^_xx_) along (i) the x–axis inside (−4.5 mm ≤ x ≤ 4.5 mm) and (ii) the circumferential direction (0° ≤ θ (see Figure 11) ≤ 360°) of the isotropic prolate spheroidal inclusions (a/b = c/b = 0.75 where b = 6 mm) under uniform remote tensile loading. It was determined in Figure 12 and Figure 13 that the normalized tensile stress (σ_xx_/σ^o^_xx_) inside the isotropic prolate spheroidal inclusions is constant in all directions considered.

#### 3.2.2. A Single Orthotropic Prolate Spheroidal Inclusion

Two different prolate spheroidal inclusions are considered: (a) a/b = c/b = 0.5, where b = 6 mm, and (b) a/b = c/b = 0.75, where b = 6 mm (see Figure 5). It should be noted that the length of b (=6 mm) can be arbitrarily chosen.

Figure 10 and Figure 11 show a typical discretized model for the single (a) prolate spheroidal inclusion (a/b = c/b = 0.5 where b = 6 mm) and (b) prolate spheroidal inclusion (a/b = c/b = 0.75 where b = 6 mm) used in the VIEM [31], respectively. A total of 7560 standard 20-node quadratic hexahedral elements were used for the single prolate spheroidal inclusion in Figure 10 and Figure 11. The number of elements, 7560, was determined based on a convergence test. 

**Figure 14:**
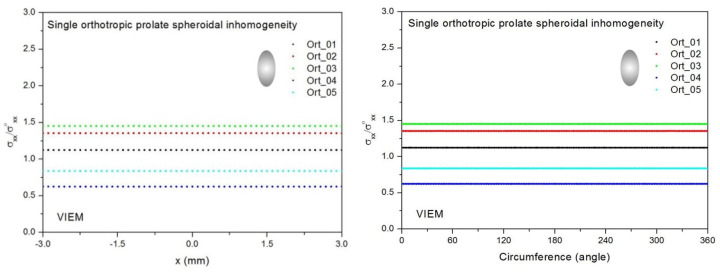
VIEM results for the normalized tensile stress component (σ_xx_/σ^o^_xx_) along (**left**) the x–axis inside and (**right**) the circumferential direction of the orthotropic prolate spheroidal inclusions (Ort_01, Ort_02, Ort_03, Ort_04 and Ort_05) with a/b = c/b = 0.5 (b = 6 mm) under uniform remote tensile loading.

Five different orthotropic inclusions (from Ort_01 to Ort_05) in Table 3 were used in the numerical calculation. It should be noted that the VIEM results represent average values of the normalized stresses in all the nodes of the VIEM model in Figure 10 and Figure 11. It should also be noted that the normalized tensile stress (σ_xx_/σ^o^_xx_) inside the orthotropic prolate spheroidal inclusions was found to be constant [1,30]. Table 13 shows numerical solutions by the volume integral equation method for the normalized tensile stress (σ_xx_/σ^o^_xx_) inside the orthotropic prolate spheroidal inclusions. For the inclusions in Ort_01, Ort_02 and Ort_03, the normalized tensile stress (σ_xx_/σ^o^_xx_) inside the inclusion was greater than 1.0. However, for the inclusions in Ort_04 and Iso_05, the normalized tensile stress (σ_xx_/σ^o^_xx_) inside the inclusion was less than 1.0. Figure 14 shows numerical solution by the volume integral equation method for the normalized tensile stress (σ_xx_/σ^o^_xx_) along (left) the x–axis inside (−3 mm ≤ x ≤ 3 mm) and (right) the circumferential direction (0° ≤ θ (see Figure 10) ≤ 360°) of the orthotropic prolate spheroidal inclusions (a/b = c/b = 0.5 where b = 6 mm) under uniform remote tensile loading.

**Figure 15:**
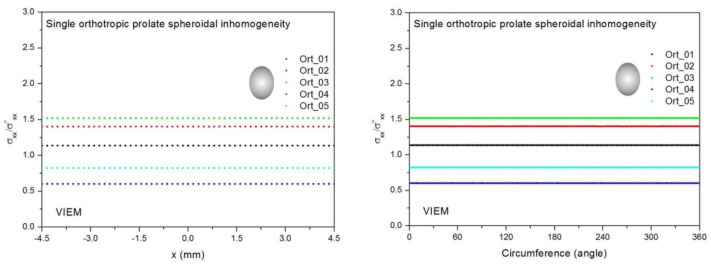
VIEM results for the normalized tensile stress component (σ_xx_/σ^o^_xx_) along (**left**) the x–axis inside and (**right**) the circumferential direction of the orthotropic prolate spheroidal inclusions (Ort_01, Ort_02, Ort_03, Ort_04 and Ort_05) with a/b = c/b = 0.75 (b = 6 mm) under uniform remote tensile loading.

Figure 15 shows numerical solutions by the volume integral equation method for the normalized tensile stress (σ_xx_/σ^o^_xx_) along (left) the x–axis inside (−4.5 mm ≤ x ≤ 4.5 mm) and (right) the circumferential direction (0° ≤ θ (see Figure 11) ≤ 360°) of the orthotropic prolate spheroidal inclusions (a/b = c/b = 0.75 where b = 6 mm) under uniform remote tensile loading. It was determined in Figure 14 and Figure 15 that the normalized tensile stress (σ_xx_/σ^o^_xx_) inside the orthotropic prolate spheroidal inclusions is constant in all directions considered.

#### 3.2.3. A Single Isotropic Oblate Spheroidal Inclusion

In this section, two different oblate spheroidal inclusions are considered: (a) b/a = c/a = 0.5, where a = 6 mm, and (b) b/a = c/a = 0.75, where a = 6 mm (see Figure 5). It should be noted that the length of a (=6 mm) can be arbitrarily chosen.

**Figure 16:**
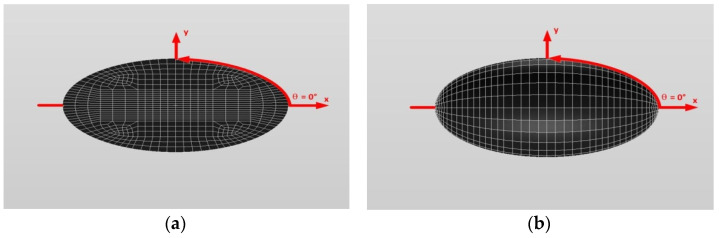
A typical discretized oblate spheroidal model (b/a = c/a = 0.5) in the volume integral equation method (VIEM). (**a**) An inside view of an oblate spheroidal model. (**b**) An oblate spheroidal model.

**Figure 17:**
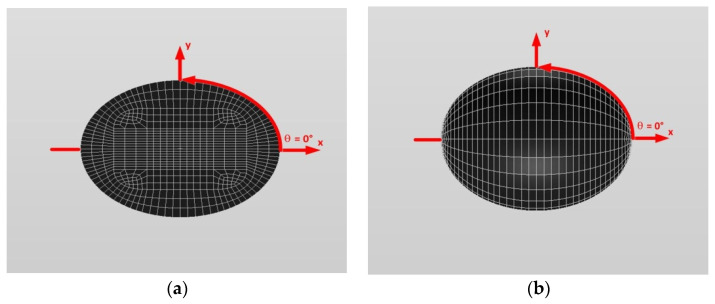
A typical discretized oblate spheroidal model (b/a = c/a = 0.75) in the volume integral equation method (VIEM). (**a**) An inside view of an oblate spheroidal model. (**b**) An oblate spheroidal model.

Figure 16 and Figure 17 show a typical discretized model for the single (a) oblate spheroidal inclusion (b/a = c/a = 0.5 where a = 6 mm) and (b) oblate spheroidal inclusion (b/a = c/a = 0.75 where a = 6 mm) used in the VIEM [31], respectively. A total of 7560 standard 20-node quadratic hexahedral elements were used for the single oblate spheroidal inclusion in Figure 16 and Figure 17. The number of elements, 7560, was determined based on a convergence test. 

**Figure 18:**
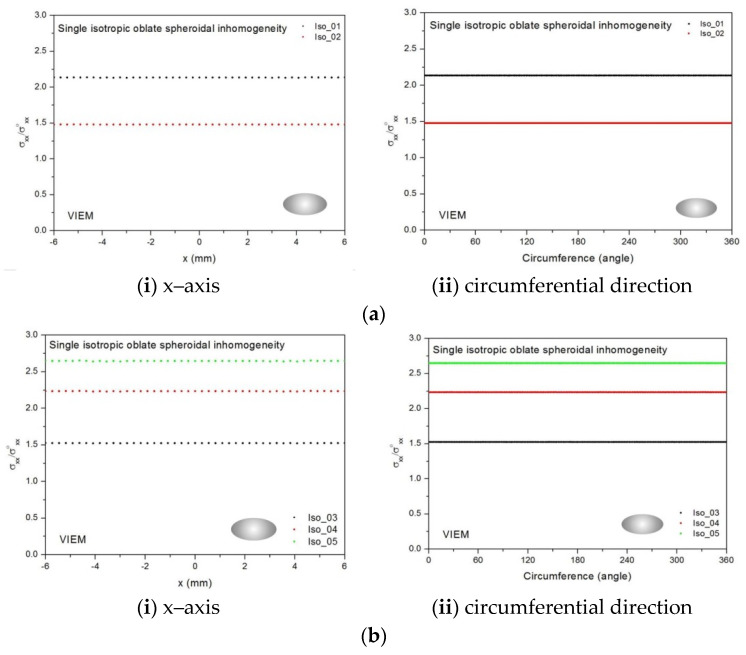

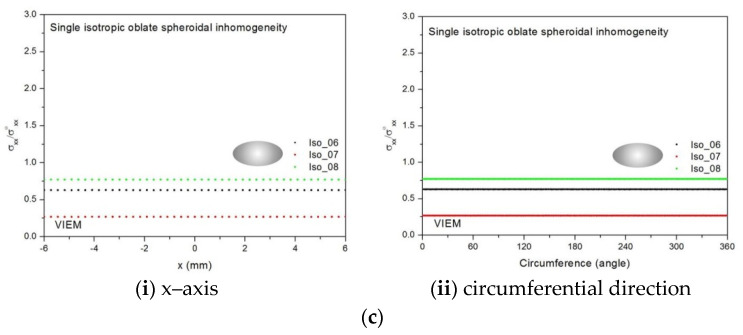
VIEM results for the normalized tensile stress component (σ_xx_/σ^o^_xx_) along (**i**) the x–axis inside and (**ii**) the circumferential direction of the isotropic oblate spheroidal inclusions with b/a = c/a = 0.5 (a = 6 mm) under uniform remote tensile loading. (**a**) Iso_01 and Iso_02. (**b**) Iso_03, Iso_04 and Iso_05. (**c**) Iso_06, Iso_07 and Iso_08.

**Figure 19:**
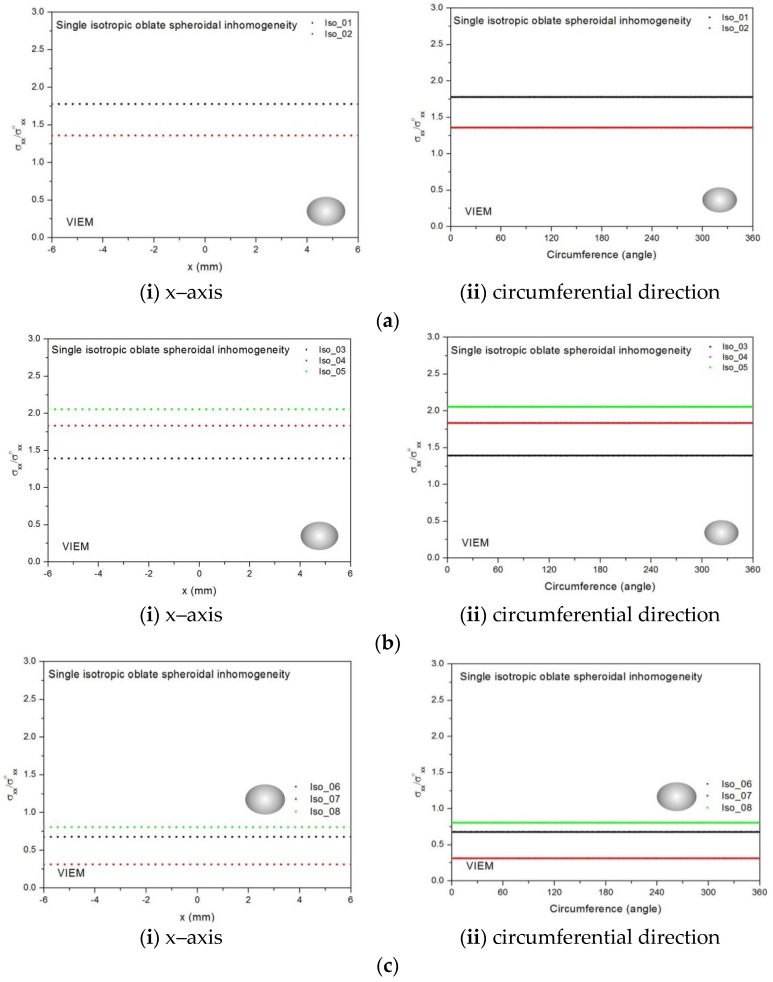
VIEM results for the normalized tensile stress component (σ_xx_/σ^o^_xx_) along (**i**) the x–axis inside and (**ii**) the circumferential direction of the isotropic oblate spheroidal inclusions with b/a = c/a = 0.75 (a = 6 mm) under uniform remote tensile loading. (**a**) Iso_01 and Iso_02. (**b**) Iso_03, Iso_04 and Iso_05. (**c**) Iso_06, Iso_07 and Iso_08.

Eight different isotropic inclusions (from Iso_01 to Iso_08) in Table 2 were used in the numerical calculation. It should be noted that the VIEM results represent average values of the normalized stresses in all the nodes of the VIEM model in Figure 16 and Figure 17. It should also be noted that the normalized tensile stress (σ_xx_/σ^o^_xx_) inside the isotropic oblate spheroidal inclusions was found to be constant [1,30]. Table 14, Table 15 and Table 16 show numerical solutions by the volume integral equation method for the normalized tensile stress (σ_xx_/σ^o^_xx_) inside the isotropic oblate spheroidal inclusions. For the inclusions in Iso_01, Iso_02, Iso_03, Iso_04 and Iso_05, the normalized tensile stress (σ_xx_/σ^o^_xx_) inside the inclusion was greater than 1.0. However, for the inclusions in Iso_06, Iso_07 and Iso_08, the normalized tensile stress (σ_xx_/σ^o^_xx_) inside the inclusion was less than 1.0. Figure 18 shows numerical solutions by the volume integral equation method for the normalized tensile stress (σ_xx_/σ^o^_xx_) along (i) the x–axis inside (−6 mm ≤ x ≤ 6 mm) and (ii) the circumferential direction (0° ≤ θ (see Figure 16) ≤ 360°) of the isotropic oblate spheroidal inclusions (b/a = c/a = 0.5 where a = 6 mm) under uniform remote tensile loading. Figure 19 shows numerical solutions by the volume integral equation method for the normalized tensile stress (σ_xx_/σ^o^_xx_) along (i) the x–axis inside (−6 mm ≤ x ≤ 6 mm) and (ii) the circumferential direction (0° ≤ θ (see Figure 17) ≤ 360°) of the isotropic oblate spheroidal inclusions (b/a = c/a = 0.75 where a = 6 mm) under uniform remote tensile loading. It was determined in Figure 18 and Figure 19 that the normalized tensile stress (σ_xx_/σ^o^_xx_) inside the isotropic oblate spheroidal inclusions is constant in all directions considered.

#### 3.2.4. A Single Orthotropic Oblate Spheroidal Inclusion

In this section, two different oblate spheroidal inclusions are considered: (a) b/a = c/a = 0.5, where a = 6 mm, and (b) b/a = c/a = 0.75, where a = 6 mm (see Figure 5). It should be noted that the length of a (=6 mm) can be arbitrarily chosen.

Figure 16 and Figure 17 show a typical discretized model for the single (a) oblate spheroidal inclusion (b/a = c/a = 0.5 where a = 6 mm) and (b) oblate spheroidal inclusion (b/a = c/a = 0.75 where a = 6 mm) used in the VIEM [31], respectively. A total of 7560 standard 20-node quadratic hexahedral elements were used for the single oblate spheroidal inclusion in Figure 16 and Figure 17. The number of elements, 7560, was determined based on a convergence test. 

**Figure 20:**
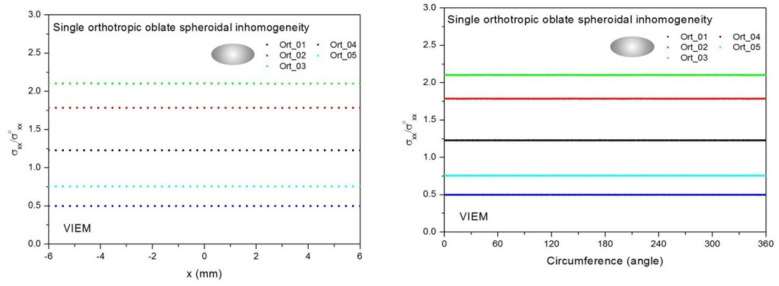
VIEM results for the normalized tensile stress component (σ_xx_/σ^o^_xx_) along (**left**) the x–axis inside and (**right**) the circumferential direction of the orthotropic oblate spheroidal inclusions (Ort_01, Ort_02, Ort_03, Ort_04 and Ort_05) with b/a = c/a = 0.5 (a = 6 mm) under uniform remote tensile loading.

**Figure 21:**
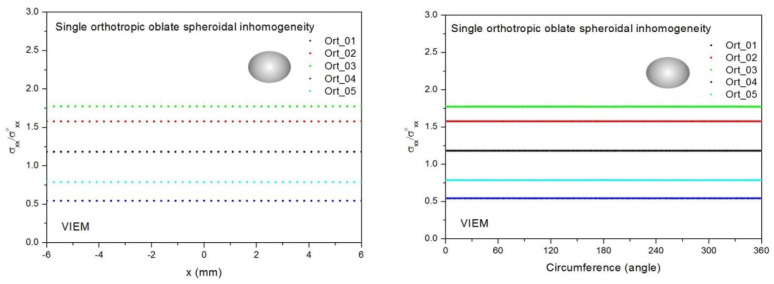
VIEM results for the normalized tensile stress component (σ_xx_/σ^o^_xx_) along (**left**) the x–axis inside and (**right**) the circumferential direction of the orthotropic oblate spheroidal inclusions (Ort_01, Ort_02, Ort_03, Ort_04 and Ort_05) with b/a = c/a = 0.75 (a = 6 mm) under uniform remote tensile loading.

Five different orthotropic inclusions (from Ort_01 to Ort_05) in Table 3 were used in the numerical calculation. It should be noted that the VIEM results represent average values of the normalized stresses in all the nodes of the VIEM model in Figure 16 and Figure 17. It should also be noted that the normalized tensile stress (σ_xx_/σ^o^_xx_) inside the orthotropic oblate spheroidal inclusions was found to be constant [1,30]. Table 17 shows numerical solutions by the volume integral equation method for the normalized tensile stress (σ_xx_/σ^o^_xx_) inside the orthotropic oblate pheroidal inclusions. For the inclusions in Ort_01, Ort_02 and Ort_03, the normalized tensile stress (σ_xx_/σ^o^_xx_) inside the inclusion was greater than 1.0. However, for the inclusions in Ort_04 and Iso_05, the normalized tensile stress (σ_xx_/σ^o^_xx_) inside the inclusion was less than 1.0. Figure 20 shows numerical solutions by the volume integral equation method for the normalized tensile stress (σ_xx_/σ^o^_xx_) along (left) the x–axis inside (−6 mm ≤ x ≤ 6 mm) and (right) the circumferential direction (0° ≤ θ (see Figure 16) ≤ 360°) of the orthotropic oblate spheroidal inclusions (b/a = c/a = 0.5 where a = 6 mm) under uniform remote tensile loading. Figure 21 shows numerical solutions by the volume integral equation method for the normalized tensile stress (σ_xx_/σ^o^_xx_) along (left) the x–axis inside (−6 mm ≤ x ≤ 6 mm) and (right) the circumferential direction (0° ≤ θ (see Figure 17) ≤ 360°) of the orthotropic oblate spheroidal inclusions (b/a = c/a = 0.75 where a = 6 mm) under uniform remote tensile loading. It was determined in Figure 20 and Figure 21 that the normalized tensile stress (σ_xx_/σ^o^_xx_) inside the orthotropic oblate spheroidal inclusions is constant in all directions considered.

From Figure 8, Figure 9, Figure 12, Figure 13, Figure 14, Figure 15, Figure 18, Figure 19, Figure 20 and Figure 21 and Table 6, Table 7, Table 8, Table 9, Table 10, Table 11, Table 12, Table 13, Table 14, Table 15, Table 16 and Table 17, it was determined that if the inclusion is harder than the matrix, the normalized tensile stress (σ_xx_/σ^o^_xx_) inside the inclusion is greater than 1.0. Additionally, the normalized tensile stress (σ_xx_/σ^o^_xx_) inside the prolate spheroidal inclusion (a/b = c/b = 0.75) is greater than that inside the prolate spheroidal inclusion (a/b = c/b = 0.5). However, the normalized tensile stress (σ_xx_/σ^o^_xx_) inside the oblate spheroidal inclusion (b/a = c/a = 0.5) is greater than that inside the oblate spheroidal inclusion (b/a = c/a = 0.75). Thus, the normalized tensile stress (σ_xx_/σ^o^_xx_) inside the inclusion can be arranged in ascending order of magnitude: (1) prolate spheroidal inclusion (a/b = c/b = 0.5), (2) prolate spheroidal inclusion (a/b = c/b = 0.75), (3) sphere, (4) oblate spheroidal inclusion (b/a = c/a = 0.75) and (5) oblate spheroidal inclusion (b/a = c/a = 0.5). From Figure 8, Figure 9, Figure 12, Figure 13, Figure 14, Figure 15, Figure 18, Figure 19, Figure 20 and Figure 21 and Table 6, Table 7, Table 8, Table 9, Table 10, Table 11, Table 12, Table 13, Table 14, Table 15, Table 16 and Table 17, it was also determined that if the inclusion is softer than the matrix, the normalized tensile stress (σ_xx_/σ^o^_xx_) inside the inclusion is less than 1.0. Additionally, the normalized tensile stress (σ_xx_/σ^o^_xx_) inside the prolate spheroidal inclusion (a/b = c/b = 0.5) is greater than that inside the prolate spheroidal inclusion (a/b = c/b = 0.75). However, the normalized tensile stress (σ_xx_/σ^o^_xx_) inside the oblate spheroidal inclusion (b/a = c/a = 0.75) is greater than that inside the oblate spheroidal inclusion (b/a = c/a = 0.5). Thus, the normalized tensile stress (σ_xx_/σ^o^_xx_) inside the inclusion can be arranged in ascending order of magnitude: (1) oblate spheroidal inclusion (b/a = c/a = 0.5), (2) oblate spheroidal inclusion (b/a = c/a = 0.75), (3) sphere, (4) prolate spheroidal inclusion (a/b = c/b = 0.75) and (5) prolate spheroidal inclusion (a/b = c/b = 0.5).

Both the standard finite element method (FEM) and the boundary element method (BEM) are powerful general-purpose tools in the field of numerical analysis. Since the VIEM is a combination of these two methods, it is also highly beneficial to the field of numerical analysis and can play a very important role in solving “inclusion problems”. The authors hope that the results using the VIEM cited in this paper will be used as benchmarked data for verifying the results of similar research using other analytical and numerical methods.

### 3.3. Single Spherical Inclusion Problems under Remote Shear Loading

#### 3.3.1. VIEM Formulation Applied to Isotropic/Orthotropic Inclusion Problems

The displacements for isotropic spherical, prolate and oblate spheroidal inclusions can be determined from volume integral Equations (3)–(5), while the displacements for orthotropic spherical, prolate and oblate spheroidal inclusions can be determined from volume integral Equations (6)–(8).

#### 3.3.2. A Single Isotropic Spherical Inclusion

**Figure 22:**
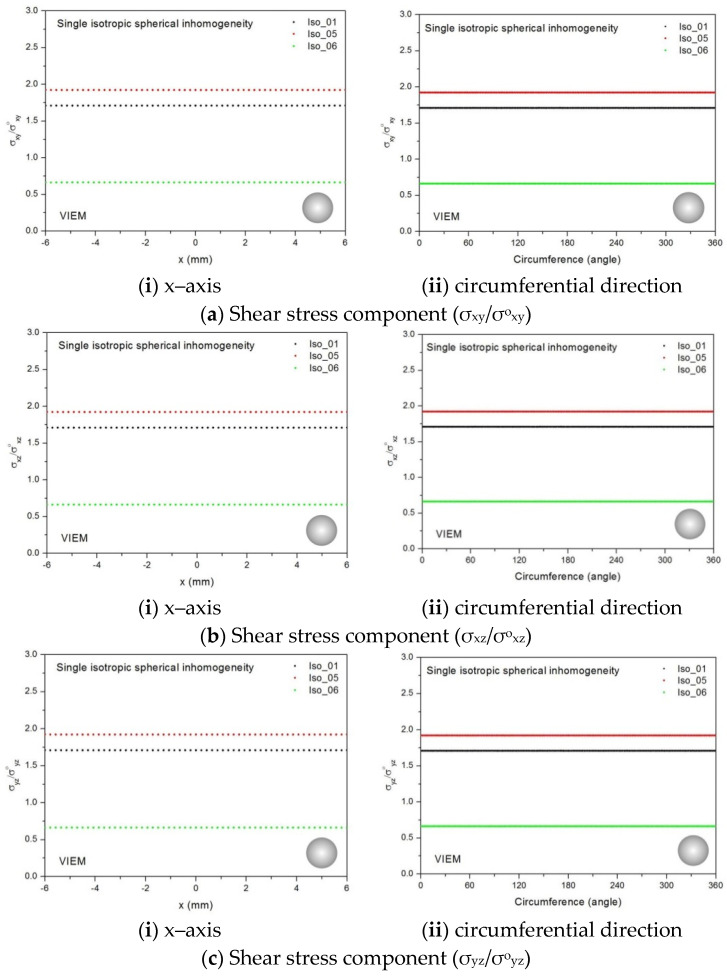
VIEM results for the normalized shear stress components (**a**) σ_x__y_/σ^o^_x__y_, (**b**) σ_x__z_/σ^o^_x__z_ and (**c**) σ_yz_/σ^o^_yz_ along (**i**) the x–axis inside and (**ii**) the circumferential direction of the isotropic spherical inclusions (Iso_01, Iso_05 and Iso_06) with a radius of 6 mm under remote shear loading (σ^o^_x__y_, σ^o^_x__z_ and σ^o^_yz_).

We considered a single isotropic spherical inclusion with a radius of 6 mm in an infinite isotropic matrix subject to remote shear loading, σ^o^_x__y_, σ^o^_x__z_ and σ^o^_yz_, as shown in Figure 6a [24]. It should be noted that the length of the radius can be arbitrarily chosen. In Figure 7, standard 20-node quadratic hexahedral elements were used in the discretization [31]. The number of hexahedral elements, 7560, was determined based on a convergence test. Three different material properties (Iso_01, Iso_05 and Iso_06) in Table 2 were used in the numerical calculation. It should be noted that the normalized shear stresses (σ_x__y_/σ^o^_x__y_, σ_x__z_/σ^o^_x__z_ and σ_yz_/σ^o^_yz_) inside the isotropic spherical inclusions were found to be constant, respectively [1]. It should also be noted that the VIEM results represent average values of the normalized stresses in all the nodes of the VIEM model in Figure 7. Table 18 shows numerical solutions by the volume integral equation method for the normalized shear stresses (σ_x__y_/σ^o^_x__y_, σ_x__z_/σ^o^_x__z_ and σ_yz_/σ^o^_yz_) inside the isotropic spherical inclusions. For the inclusions in Iso_01 and Iso_05, the normalized shear stresses (σ_x__y_/σ^o^_x__y_, σ_x__z_/σ^o^_x__z_ and σ_yz_/σ^o^_yz_) inside the inclusion were greater than 1.0, respectively. However, for the inclusion in Iso_06, the normalized shear stresses (σ_x__y_/σ^o^_x__y_, σ_x__z_/σ^o^_x__z_ and σ_yz_/σ^o^_yz_) inside the inclusion were less than 1.0, respectively. Figure 22 shows numerical solutions by the volume integral equation method for the normalized shear stresses (σ_x__y_/σ^o^_x__y_, σ_x__z_/σ^o^_x__z_ and σ_yz_/σ^o^_yz_) along (i) the x–axis inside (−6 mm ≤ x ≤ 6 mm) and (ii) the circumferential direction (0° ≤ θ (see Figure 7) ≤ 360°) of the isotropic spherical inclusions with a radius of 6 mm under remote shear loading. 

In most references, spherical inclusion problems under uniform remote tensile loading were considered. Thus, in order to show the VIEM results more thoroughly, the normalized shear stresses, (a) σ_x__y_/σ^o^_x__y_, (b) σ_x__z_/σ^o^_x__z_ and (c) σ_yz_/σ^o^_yz_, using the VIEM were presented along (i) the x–axis inside (−6 mm ≤ x ≤ 6 mm) and (ii) the circumferential direction (0° ≤ θ (see Figure 7) ≤ 360°) of the isotropic spherical inclusions. 

It was determined in Figure 22 that the normalized shear stresses (σ_x__y_/σ^o^_x__y_, σ_x__z_/σ^o^_x__z_ and σ_yz_/σ^o^_yz_) inside the single isotropic spherical inclusions are constant in all directions considered and are identical to each other. Since isotropic materials have an infinite number of planes of symmetry, the normalized shear stresses (σ_x__y_/σ^o^_x__y_, σ_x__z_/σ^o^_x__z_ and σ_yz_/σ^o^_yz_) inside the single isotropic spherical inclusions turned out to be identical to each other.

#### 3.3.3. A Single Orthotropic Spherical Inclusion

In order to show the advantages of the volume integral equation method (VIEM), we considered a single orthotropic spherical inclusion with a radius of 6 mm in an infinite isotropic matrix subject to remote shear loading, σ^o^_x__y_, σ^o^_x__z_ and σ^o^_yz_, as shown in Figure 6a. It should be noted that the length of the radius can be arbitrarily chosen. In Figure 7, standard 20-node quadratic hexahedral elements were used in the discretization [31]. The number of hexahedral elements was 7560, determined based on a convergence test. For this problem, in comparison to the boundary element method (BEM), since the VIEM is not sensitive to the anisotropy of the inclusions, it does not require the use of the Green’s function for the anisotropic inclusions. Moreover, as opposed to the standard FEM, where it is necessary to discretize the full domain, the orthotropic inclusion only needs to be discretized in the VIEM. 

**Figure 23:**
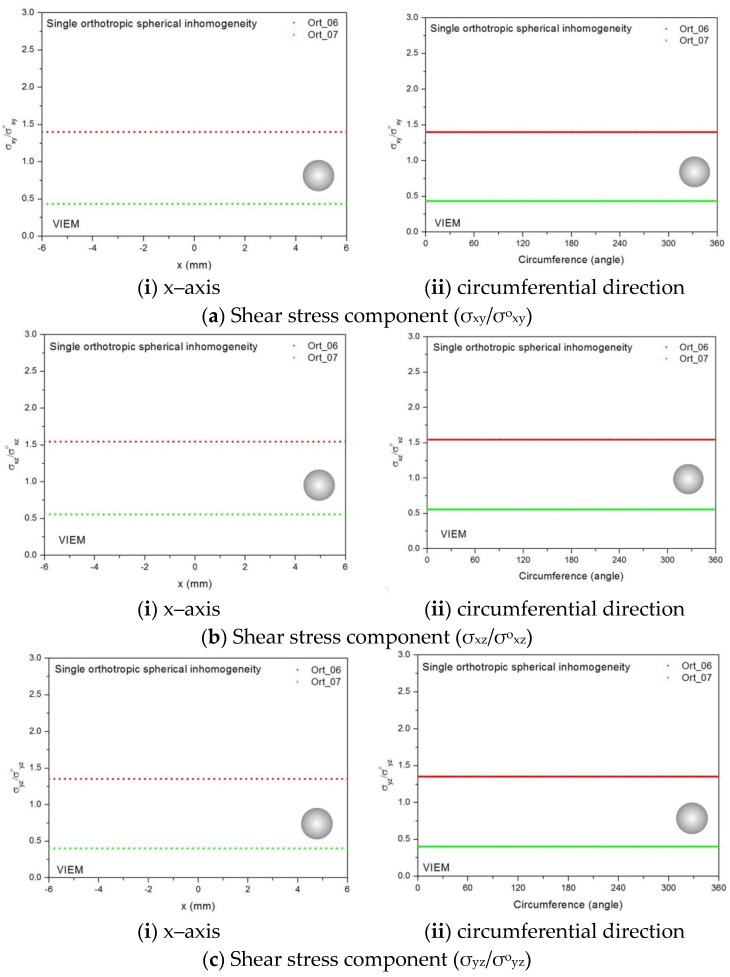
VIEM results for the normalized shear stress components (**a**) σ_x__y_/σ^o^_x__y_, (**b**) σ_x__z_/σ^o^_x__z_ and (**c**) σ_yz_/σ^o^_yz_ along (**i**) the x–axis inside and (**ii**) the circumferential direction of the orthotropic spherical inclusions (Ort_06 and Ort_07) with a radius of 6 mm under remote shear loading (σ^o^_x__y_, σ^o^_x__z_ and σ^o^_yz_).

Two different material properties (Ort_06 and Ort_07) in Table 4 were used in the numerical calculation [25]. As shown in Table 5, it was assumed that c_55_ > c_66_ > c_44_ for two orthotropic inclusions. Additionally, c_44_, c_55_ and c_66_ of the inclusion were assumed be greater than μ of the matrix in the Ort_06 material, while μ of the matrix was assumed to be greater than c_44_, c_55_ and c_66_ of the inclusion in the Ort_07 material. Thus, two material properties representing different characteristics were chosen. It should be noted that the VIEM results represent average values of the normalized stresses in all the nodes of the VIEM model in Figure 7. Moreover, the normalized shear stresses (σ_x__y_/σ^o^_x__y_, σ_x__z_/σ^o^_x__z_ and σ_yz_/σ^o^_yz_) inside the orthotropic spherical inclusions were found to be constant, respectively [1]. Table 19 shows numerical solutions by the volume integral equation method for the normalized shear stresses (σ_x__y_/σ^o^_x__y_, σ_x__z_/σ^o^_x__z_ and σ_yz_/σ^o^_yz_) inside the orthotropic spherical inclusions. For the inclusion in Ort_06, the normalized shear stresses (σ_x__y_/σ^o^_x__y_, σ_x__z_/σ^o^_x__z_ and σ_yz_/σ^o^_yz_) inside the inclusion were greater than 1.0, respectively. However, for the inclusion in Ort_07, the normalized shear stresses (σ_x__y_/σ^o^_x__y_, σ_x__z_/σ^o^_x__z_ and σ_yz_/σ^o^_yz_) inside the inclusion were less than 1.0, respectively. Figure 23 shows numerical solutions by the volume integral equation method for the normalized shear stresses (a) σ_x__y_/σ^o^_x__y_, (b) σ_x__z_/σ^o^_x__z_ and (c) σ_yz_/σ^o^_yz_ along (i) the x–axis inside (−6 mm ≤ x ≤ 6 mm) and (ii) the circumferential direction (0° ≤ θ (see Figure 7) ≤ 360°) of the orthotropic spherical inclusions with a radius of 6 mm under remote shear loading. It was determined in Figure 23 that the normalized shear stresses (σ_x__y_/σ^o^_x__y_, σ_x__z_/σ^o^_x__z_ and σ_yz_/σ^o^_yz_) inside the orthotropic spherical inclusions are constant in all directions considered and are different from each other. Since orthotropic materials have three planes/axes of symmetry and the independent shear moduli in three planes of symmetry are different from each other (c_55_ > c_66_ > c_44_), the normalized shear stresses (σ_x__y_/σ^o^_x__y_, σ_x__z_/σ^o^_x__z_ and σ_yz_/σ^o^_yz_) inside the orthotropic spherical inclusions turned out to be different from each other. Furthermore, since c_55_ (shear modulus in the xz plane) is greater than c_66_ (shear modulus in the xy plane) and c_66_ is greater than c_44_ (shear modulus in the yz plane) in the orthotropic inclusions of the Ort_06 and Ort_07 materials, it was determined that the normalized shear stress, σ_x__z_/σ^o^_x__z_, was greater than the normalized shear stress, σ_x__y_/σ^o^_x__y_. Furthermore, σ_x__y_/σ^o^_x__y_ was found to be greater than the normalized shear stress, σ_yz_/σ^o^_yz_, inside the orthotropic spherical inclusions.

### 3.4. A Single Spheroidal Inclusion Problem under Remote Shear Loading

In order to introduce the VIEM as a versatile numerical method, we considered a single isotropic/orthotropic spheroidal inclusion in an infinite isotropic matrix subject to remote shear loading, σ^o^_x__y_, σ^o^_x__z_ and σ^o^_yz_, as shown in Figure 6b,c. Figure 5 shows the orientation of the spheroidal inclusion.

#### 3.4.1. A Single Isotropic Prolate Spheroidal Inclusion

Two different prolate spheroidal inclusions are considered: (a) a/b = c/b = 0.5, where b = 6 mm, and (b) a/b = c/b = 0.75, where b = 6 mm (see Figure 5). It should be noted that the length of b (=6 mm) can be arbitrarily chosen.

Figure 10 and Figure 11 show a typical discretized model for the single (a) prolate spheroidal inclusion (a/b = c/b = 0.5 where b = 6 mm) and (b) prolate spheroidal inclusion (a/b = c/b = 0.75 where b = 6 mm) used in the VIEM [31], respectively. A total of 7560 standard 20-node quadratic hexahedral elements were used for the single prolate spheroidal inclusion in Figure 10 and Figure 11. The number of elements, 7560, was determined based on a convergence test. 

**Figure 24:**
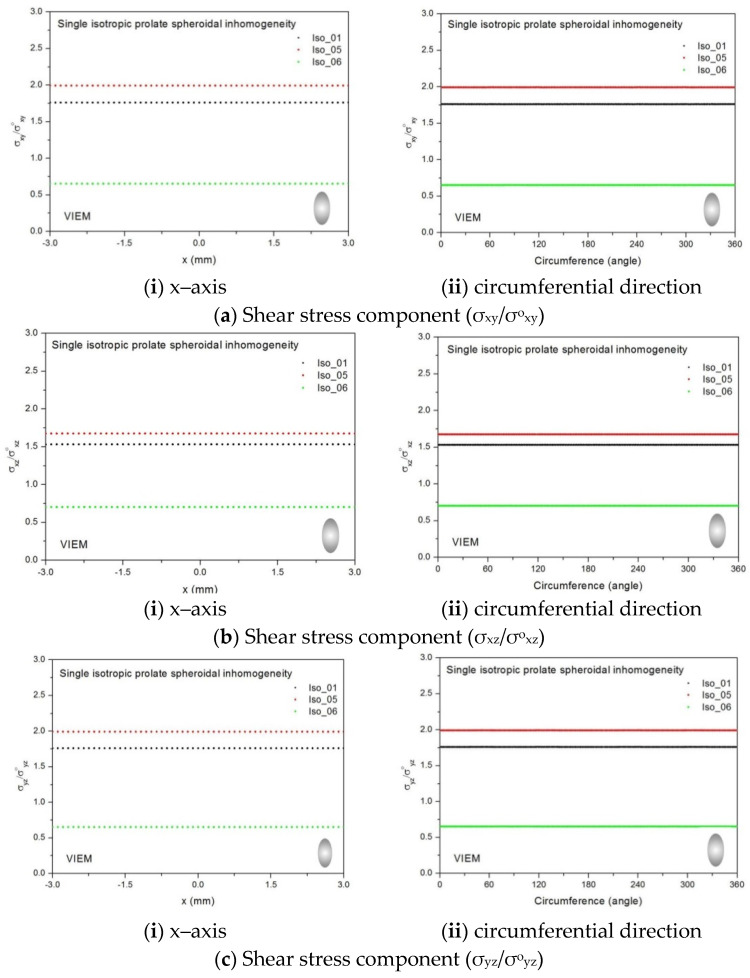
VIEM results for the normalized shear stress components (**a**) σ_x__y_/σ^o^_x__y_, (**b**) σ_x__z_/σ^o^_x__z_ and (**c**) σ_yz_/σ^o^_yz_ along (**i**) the x–axis inside and (**ii**) the circumferential direction of the isotropic prolate spheroidal inclusions (Iso_01, Iso_05 and Iso_06) with a/b = c/b = 0.5 (b = 6 mm) under remote shear loading (σ^o^_x__y_, σ^o^_x__z_ and σ^o^_yz_).

**Figure 25:**
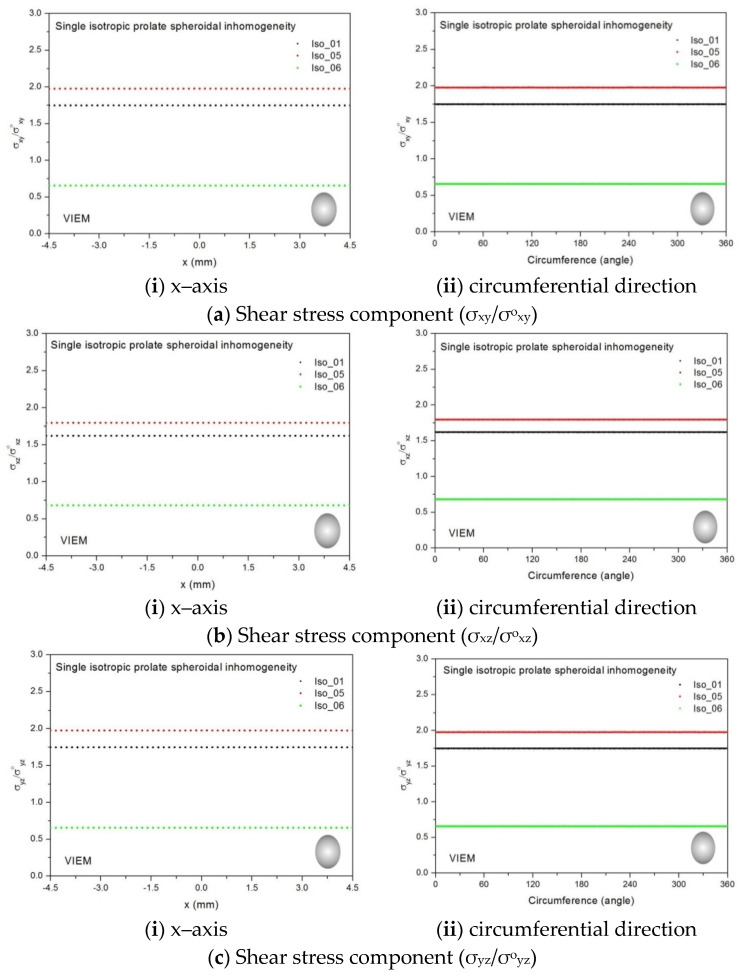
VIEM results for the normalized shear stress components (**a**) σ_x__y_/σ^o^_x__y_, (**b**) σ_x__z_/σ^o^_x__z_ and (**c**) σ_yz_/σ^o^_yz_ along (**i**) the x–axis inside and (**ii**) the circumferential direction of the isotropic prolate spheroidal inclusions (Iso_01, Iso_05 and Iso_06) with a/b = c/b = 0.75 (b = 6 mm) under remote shear loading (σ^o^_x__y_, σ^o^_x__z_ and σ^o^_yz_).

**Figure 26:**
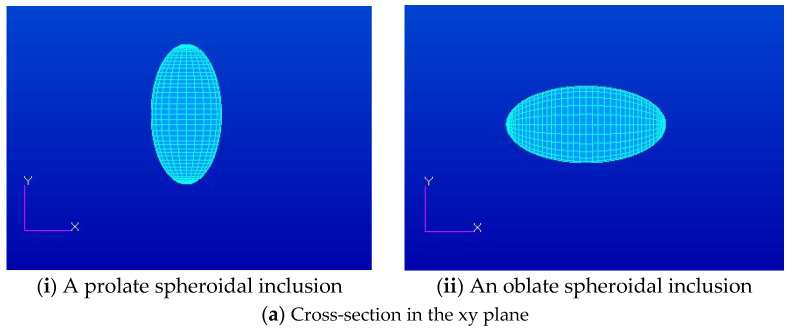

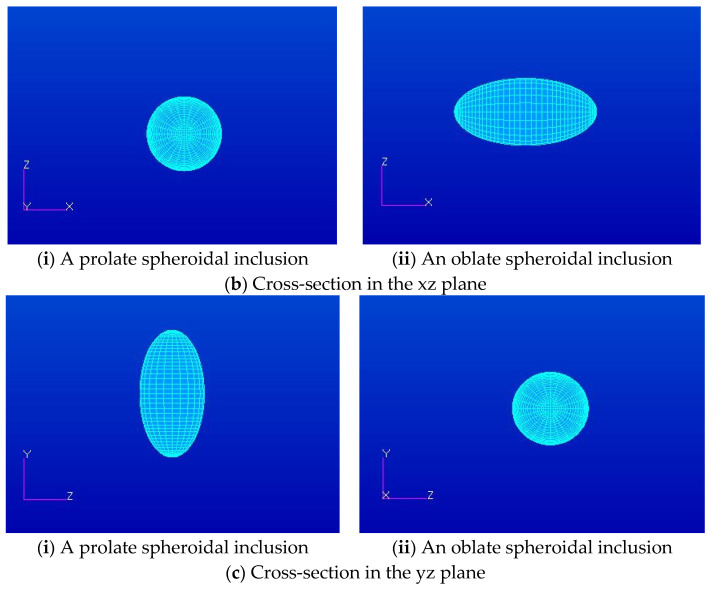
Cross-section in the (**a**) xy plane, (**b**) xz plane and (**c**) yz plane of (**i**) prolate spheroidal (with an aspect ratio of 0.5) and (**ii**) oblate spheroidal (with an aspect ratio of 0.5) inclusions under remote shear loading.

Three different isotropic inclusions (Iso_01, Iso_05 and Iso_06) in Table 2 were used in the numerical calculation. It should be noted that the VIEM results represent average values of the normalized stresses in all the nodes of the VIEM model in Figure 10 and Figure 11. It should also be noted that the normalized shear stresses (σ_x__y_/σ^o^_x__y_, σ_x__z_/σ^o^_x__z_ and σ_yz_/σ^o^_yz_) inside the isotropic prolate spheroidal inclusions were found to be constant, respectively [1]. Table 20 shows numerical solutions by the volume integral equation method for the normalized shear stresses (σ_x__y_/σ^o^_x__y_, σ_x__z_/σ^o^_x__z_ and σ_yz_/σ^o^_yz_) inside the isotropic prolate spheroidal inclusions. For the inclusions in Iso_01 and Iso_05, the normalized shear stresses (σ_x__y_/σ^o^_x__y_, σ_x__z_/σ^o^_x__z_ and σ_yz_/σ^o^_yz_) inside the inclusion were greater than 1.0, respectively. However, for the inclusion in Iso_06, the normalized shear stresses (σ_x__y_/σ^o^_x__y_, σ_x__z_/σ^o^_x__z_ and σ_yz_/σ^o^_yz_) inside the inclusion were less than 1.0, respectively. Figure 24 shows numerical solutions by the volume integral equation method for the normalized shear stresses (a) σ_x__y_/σ^o^_x__y_, (b) σ_x__z_/σ^o^_x__z_ and (c) σ_yz_/σ^o^_yz_ along (i) the x–axis inside (−3 mm ≤ x ≤ 3 mm) and (ii) the circumferential direction (0° ≤ θ (see Figure 10) ≤ 360°) of the isotropic prolate spheroidal inclusions (a/b = c/b = 0.5 where b = 6 mm) under remote shear loading, σ^o^_x__y_, σ^o^_x__z_ and σ^o^_yz_. Figure 25 shows numerical solutions by the volume integral equation method for the normalized shear stresses (a) σ_x__y_/σ^o^_x__y_, (b) σ_x__z_/σ^o^_x__z_ and (c) σ_yz_/σ^o^_yz_ along (i) the x–axis inside (−4.5 mm ≤ x ≤ 4.5 mm) and (ii) the circumferential direction (0° ≤ θ (see Figure 11) ≤ 360°) of the isotropic prolate spheroidal inclusions (a/b = c/b = 0.75 where b = 6 mm) under remote shear loading, σ^o^_x__y_, σ^o^_x__z_ and σ^o^_yz_. It was determined in Figure 24 and Figure 25 that the normalized shear stresses (σ_x__y_/σ^o^_x__y_, σ_x__z_/σ^o^_x__z_ and σ_yz_/σ^o^_yz_) inside the isotropic prolate spheroidal inclusions are constant in all directions considered. Furthermore, since, as shown in Figure 26, the cross-section in the xy plane is identical to the cross-section in the yz plane in the prolate spheroidal inclusion, the normalized shear stress, σ_x__y_/σ^o^_x__y_, was identical to the normalized shear stress, σ_yz_/σ^o^_yz_, inside the isotropic prolate spheroidal inclusion under remote shear loading.

#### 3.4.2. A Single Orthotropic Prolate Spheroidal Inclusion

Two different prolate spheroidal inclusions are considered: (a) a/b = c/b = 0.5, where b = 6 mm, and (b) a/b = c/b = 0.75, where b = 6 mm (see Figure 5). It should be noted that the length of b (=6 mm) can be arbitrarily chosen.

Figure 10 and Figure 11 show a typical discretized model for the single (a) prolate spheroidal inclusion (a/b = c/b = 0.5 where b = 6 mm) and (b) prolate spheroidal inclusion (a/b = c/b = 0.75 where b = 6 mm) used in the VIEM [31], respectively. A total of 7560 standard 20-node quadratic hexahedral elements were used for the single prolate spheroidal inclusion in Figure 10 and Figure 11. The number of elements, 7560, was determined based on a convergence test. 

**Figure 27:**
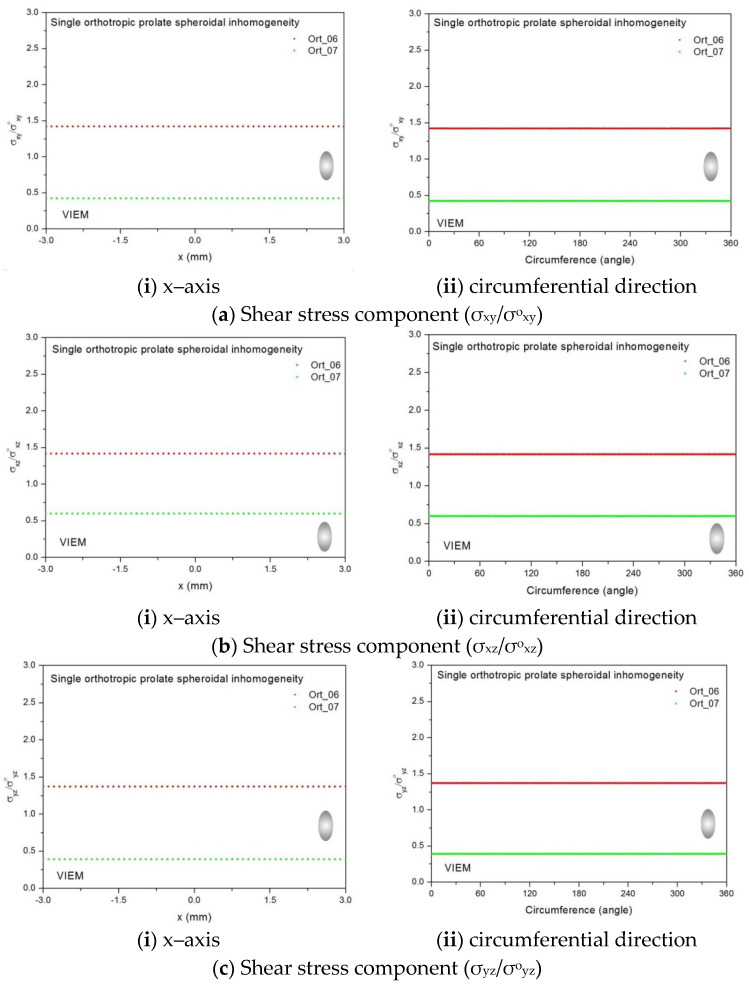
VIEM results for the normalized shear stress components (**a**) σ_x__y_/σ^o^_x__y_, (**b**) σ_x__z_/σ^o^_x__z_ and (**c**) σ_yz_/σ^o^_yz_ along (**i**) the x–axis inside and (**ii**) the circumferential direction of the orthotropic prolate spheroidal inclusions (Ort_06 and Ort_07) with a/b = c/b = 0.5 (b = 6 mm) under remote shear loading (σ^o^_x__y_, σ^o^_x__z_ and σ^o^_yz_).

**Figure 28:**
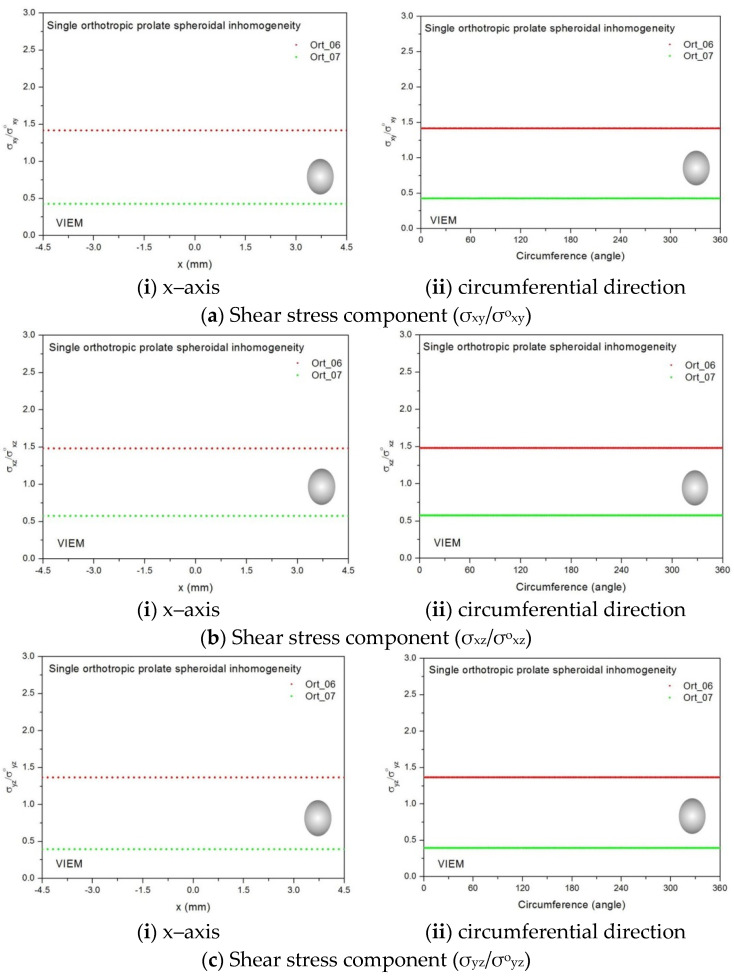
VIEM results for the normalized shear stress components (**a**) σ_x__y_/σ^o^_x__y_, (**b**) σ_x__z_/σ^o^_x__z_ and (**c**) σ_yz_/σ^o^_yz_ along (**i**) the x–axis inside and (**ii**) the circumferential direction of the orthotropic prolate spheroidal inclusions (Ort_06 and Ort_07) with a/b = c/b = 0.75 (b = 6 mm) under remote shear loading (σ^o^_x__y_, σ^o^_x__z_ and σ^o^_yz_).

Two different orthotropic inclusions (Ort_06 and Ort_07) in Table 4 were used in the numerical calculation. It should be noted that the VIEM results represent average values of the normalized stresses in all the nodes of the VIEM model in Figure 10 and Figure 11. It should also be noted that the normalized shear stresses (σ_x__y_/σ^o^_x__y_, σ_x__z_/σ^o^_x__z_ and σ_yz_/σ^o^_yz_) inside the orthotropic prolate spheroidal inclusions were found to be constant, respectively [1]. Table 21 shows numerical solutions by the volume integral equation method for the normalized shear stresses (σ_x__y_/σ^o^_x__y_, σ_x__z_/σ^o^_x__z_ and σ_yz_/σ^o^_yz_) inside the orthotropic prolate spheroidal inclusions. For the inclusion in Ort_06, the normalized shear stresses (σ_x__y_/σ^o^_x__y_, σ_x__z_/σ^o^_x__z_ and σ_yz_/σ^o^_yz_) inside the inclusion were greater than 1.0, respectively. However, for the inclusion in Ort_07, the normalized shear stresses (σ_x__y_/σ^o^_x__y_, σ_x__z_/σ^o^_x__z_ and σ_yz_/σ^o^_yz_) inside the inclusion were less than 1.0, respectively. Figure 27 shows numerical solutions by the volume integral equation method for the normalized shear stresses (a) σ_x__y_/σ^o^_x__y_, (b) σ_x__z_/σ^o^_x__z_ and (c) σ_yz_/σ^o^_yz_ along (i) the x–axis inside (−3 mm ≤ x ≤ 3 mm) and (ii) the circumferential direction (0° ≤ θ (see Figure 10) ≤ 360°) of the orthotropic prolate spheroidal inclusions (a/b = c/b = 0.5 where b = 6 mm) under remote shear loading, σ^o^_x__y_, σ^o^_x__z_ and σ^o^_yz_. Figure 28 shows numerical solutions by the volume integral equation method for the normalized shear stresses (a) σ_x__y_/σ^o^_x__y_, (b) σ_x__z_/σ^o^_x__z_ and (c) σ_yz_/σ^o^_yz_ along (i) the x–axis inside (−4.5 mm ≤ x ≤ 4.5 mm) and (ii) the circumferential direction (0° ≤ θ (see Figure 11) ≤ 360°) of the orthotropic prolate spheroidal inclusions (a/b = c/b = 0.75 where b = 6 mm) under remote shear loading, σ^o^_x__y_, σ^o^_x__z_ and σ^o^_yz_. It was determined in Figure 27 and Figure 28 that the normalized shear stresses (σ_x__y_/σ^o^_x__y_, σ_x__z_/σ^o^_x__z_ and σ_yz_/σ^o^_yz_) inside the orthotropic prolate spheroidal inclusions are constant in all directions considered. Furthermore, even though, as shown in Figure 26, the cross-section in the xy plane is identical to the cross-section in the yz plane in the prolate spheroidal inclusion, since c_55_ (shear modulus in the xz plane) is greater than c_66_ (shear modulus in the xy plane) and c_66_ is greater than c_44_ (shear modulus in the yz plane) in the orthotropic inclusions of the Ort_06 and Ort_07 materials, the normalized shear stress, σ_x__y_/σ^o^_x__y_, was different from the normalized shear stress, σ_yz_/σ^o^_yz_, inside the isotropic prolate spheroidal inclusion under remote shear loading.

#### 3.4.3. A Single Isotropic Oblate Spheroidal Inclusion

In this section, two different oblate spheroidal inclusions are considered: (a) b/a = c/a = 0.5, where a = 6 mm, and (b) b/a = c/a = 0.75, where a = 6 mm (see Figure 5). It should be noted that the length of a (=6 mm) can be arbitrarily chosen.

Figure 16 and Figure 17 show a typical discretized model for the single (a) oblate spheroidal inclusion (b/a = c/a = 0.5 where a = 6 mm) and (b) oblate spheroidal inclusion (b/a = c/a = 0.75 where a = 6 mm) used in the VIEM [31], respectively. A total of 7560 standard 20-node quadratic hexahedral elements were used for the single oblate spheroidal inclusion in Figure 16 and Figure 17. The number of elements, 7560, was determined based on a convergence test. 

Three different isotropic inclusions (Iso_01, Iso_05 and Iso_06) in Table 2 were used in the numerical calculation. It should be noted that the VIEM results represent average values of the normalized stresses in all the nodes of the VIEM model in Figure 16 and Figure 17. It should also be noted that the normalized shear stresses (σ_x__y_/σ^o^_x__y_, σ_x__z_/σ^o^_x__z_ and σ_yz_/σ^o^_yz_) inside the isotropic oblate spheroidal inclusions were found to be constant, respectively [1]. Table 22 shows numerical solutions by the volume integral equation method for the normalized shear stresses (σ_x__y_/σ^o^_x__y_, σ_x__z_/σ^o^_x__z_ and σ_yz_/σ^o^_yz_) inside the isotropic oblate spheroidal inclusions. For the inclusions in Iso_01 and Iso_05, the normalized shear stresses (σ_x__y_/σ^o^_x__y_, σ_x__z_/σ^o^_x__z_ and σ_yz_/σ^o^_yz_) inside the inclusion were greater than 1.0, respectively. However, for the inclusion in Iso_06, the normalized shear stresses (σ_x__y_/σ^o^_x__y_, σ_x__z_/σ^o^_x__z_ and σ_yz_/σ^o^_yz_) inside the inclusion were less than 1.0, respectively.

**Figure 29:**
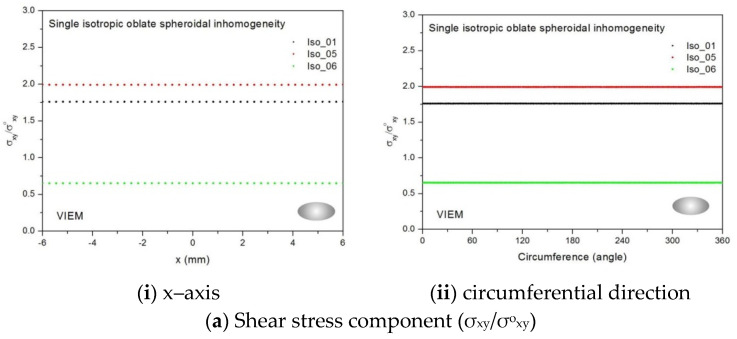

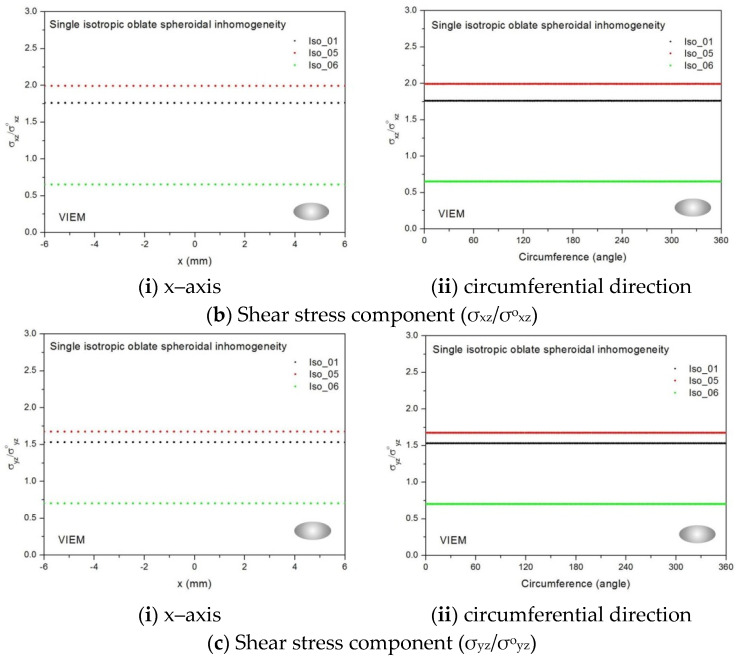
VIEM results for the normalized shear stress components (**a**) σ_x__y_/σ^o^_x__y_, (**b**) σ_x__z_/σ^o^_x__z_ and (**c**) σ_yz_/σ^o^_yz_ along (**i**) the x–axis inside and (**ii**) the circumferential direction of the isotropic oblate spheroidal inclusions (Iso_01, Iso_05 and Iso_06) with b/a = c/a = 0.5 (a = 6 mm) under remote shear loading (σ^o^_x__y_, σ^o^_x__z_ and σ^o^_yz_).

**Figure 30:**
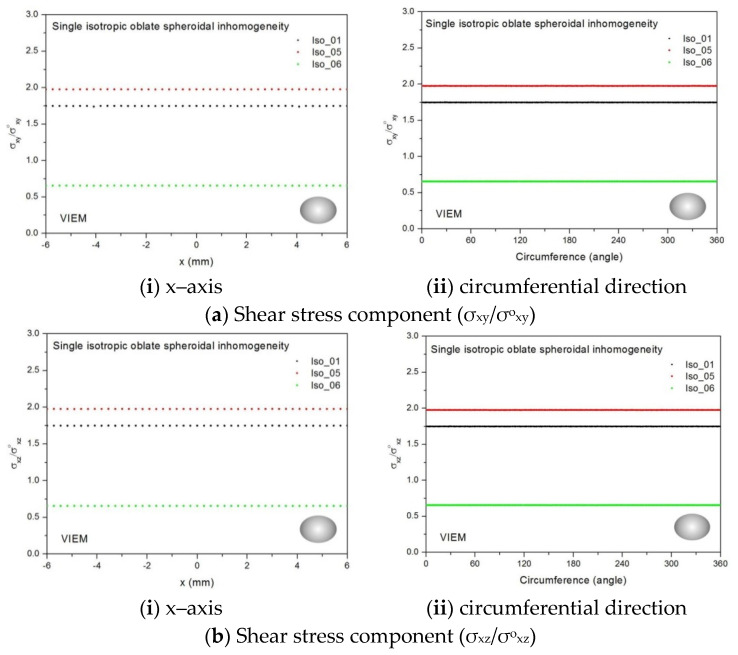

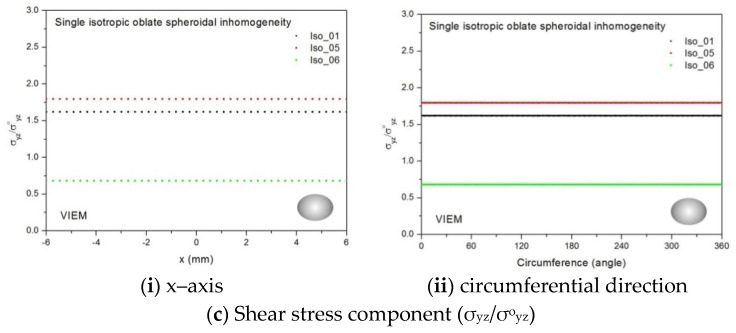
VIEM results for the normalized shear stress components (**a**) σ_x__y_/σ^o^_x__y_, (**b**) σ_x__z_/σ^o^_x__z_ and (**c**) σ_yz_/σ^o^_yz_ along (**i**) the x–axis inside and (**ii**) the circumferential direction of the isotropic oblate spheroidal inclusions (Iso_01, Iso_05 and Iso_06) with b/a = c/a = 0.75 (a = 6 mm) under remote shear loading (σ^o^_x__y_, σ^o^_x__z_ and σ^o^_yz_).

Figure 29 shows numerical results using the volume integral equation method (VIEM) for the normalized shear stresses (a) σ_x__y_/σ^o^_x__y_, (b) σ_x__z_/σ^o^_x__z_ and (c) σ_yz_/σ^o^_yz_ along (i) the x–axis inside (−6 mm ≤ x ≤ 6 mm) and (ii) the circumferential direction (0° ≤ θ (see Figure 16) ≤ 360°) of the isotropic oblate spheroidal inclusions (b/a = c/a = 0.5 where a = 6 mm) under remote shear loading, σ^o^_x__y_, σ^o^_x__z_ and σ^o^_yz_. Figure 30 shows numerical solutions by the volume integral equation method for the normalized shear stresses (a) σ_x__y_/σ^o^_x__y_, (b) σ_x__z_/σ^o^_x__z_ and (c) σ_yz_/σ^o^_yz_ along (i) the x–axis inside (−6 mm ≤ x ≤ 6 mm) and (ii) the circumferential direction (0° ≤ θ (see Figure 17) ≤ 360°) of the isotropic oblate spheroidal inclusions (b/a = c/a = 0.75 where a = 6 mm) under remote shear loading, σ^o^_x__y_, σ^o^_x__z_ and σ^o^_yz_. It was determined in Figure 29 and Figure 30 that the normalized shear stresses (σ_x__y_/σ^o^_x__y_, σ_x__z_/σ^o^_x__z_ and σ_yz_/σ^o^_yz_) inside the isotropic oblate spheroidal inclusions are constant in all directions considered. Furthermore, since, as shown in Figure 26, the cross-section in the xy plane is identical to the cross-section in the xz plane in the oblate spheroidal inclusion, the normalized shear stress, σ_x__y_/σ^o^_x__y_, was identical to the normalized shear stress, σ_xz_/σ^o^_xz_, inside the isotropic oblate spheroidal inclusion under remote shear loading.

#### 3.4.4. A Single Orthotropic Oblate Spheroidal Inclusion

In this section, two different oblate spheroidal inclusions are considered: (a) b/a = c/a = 0.5, where a = 6 mm, and (b) b/a = c/a = 0.75, where a = 6 mm (see Figure 5). It should be noted that the length of a (=6 mm) can be arbitrarily chosen. Figure 16 and Figure 17 show a typical discretized model for the single (a) oblate spheroidal inclusion (b/a = c/a = 0.5 where a = 6 mm) and (b) oblate spheroidal inclusion (b/a = c/a = 0.75 where a = 6 mm) used in the VIEM [31], respectively. A total of 7560 standard 20-node quadratic hexahedral elements were used for the single oblate spheroidal inclusion in Figure 16 and Figure 17. The number of elements, 7560, was determined based on a convergence test. 

**Figure 31:**
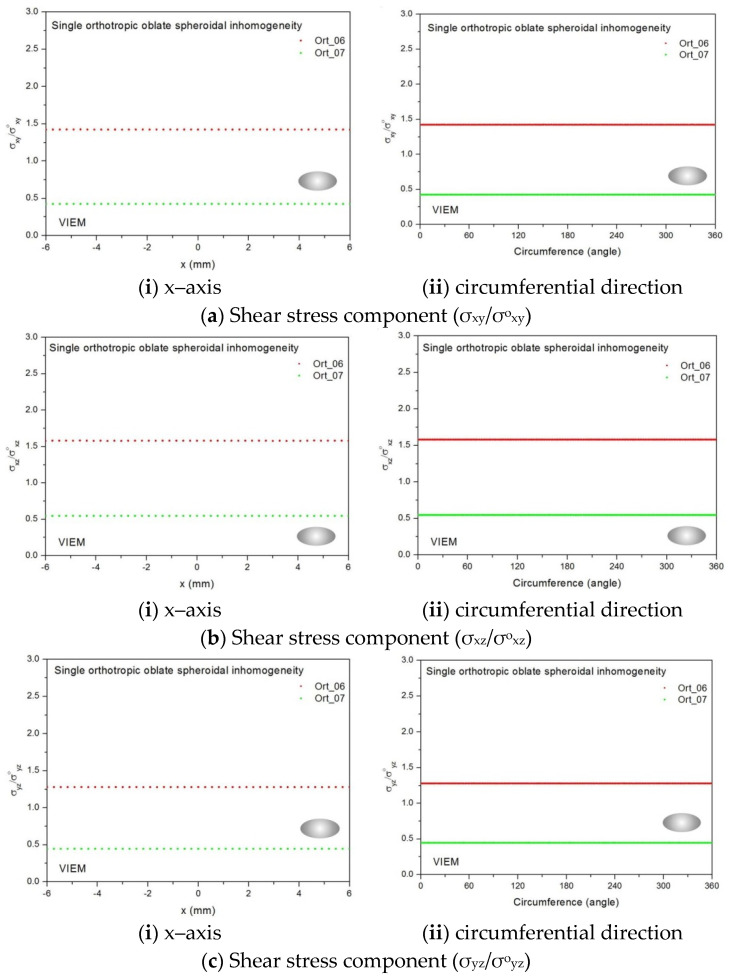
VIEM results for the normalized shear stress components (**a**) σ_x__y_/σ^o^_x__y_, (**b**) σ_x__z_/σ^o^_x__z_ and (**c**) σ_yz_/σ^o^_yz_ along (**i**) the x–axis inside and (**ii**) the circumferential direction of the orthotropic oblate spheroidal inclusions (Ort_06 and Ort_07) with b/a = c/a = 0.5 (a = 6 mm) under remote shear loading (σ^o^_x__y_, σ^o^_x__z_ and σ^o^_yz_).

**Figure 32:**
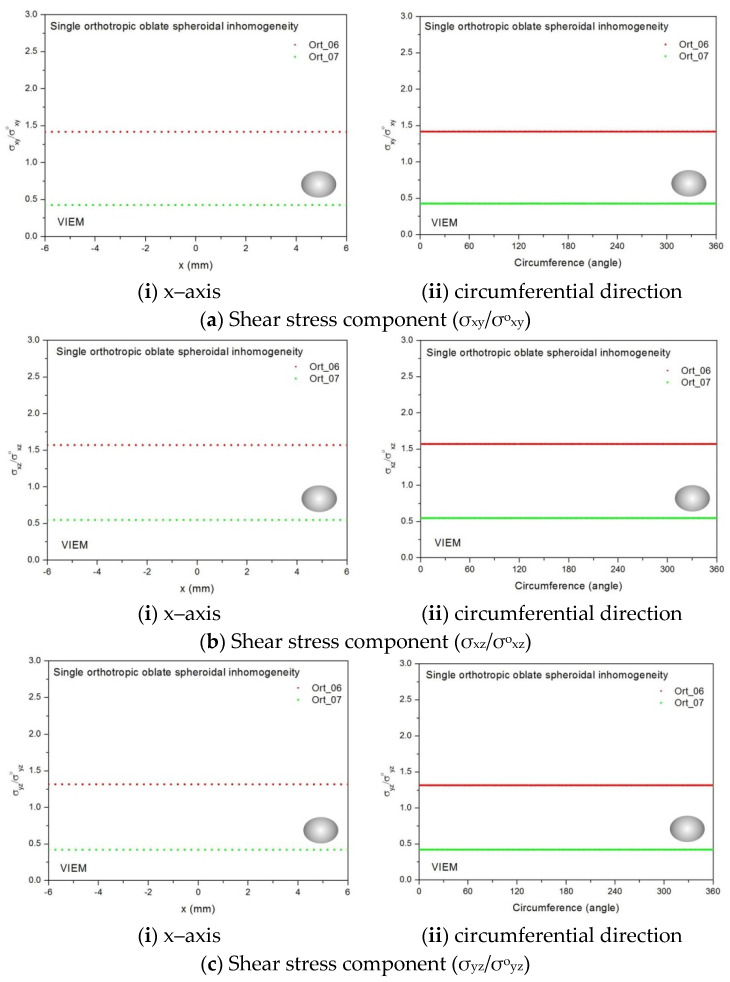
VIEM results for the normalized shear stress components (**a**) σ_x__y_/σ^o^_x__y_, (**b**) σ_x__z_/σ^o^_x__z_ and (**c**) σ_yz_/σ^o^_yz_ along (**i**) the x–axis inside and (**ii**) the circumferential direction of the orthotropic oblate spheroidal inclusions (Ort_06 and Ort_07) with b/a = c/a = 0.75 (a = 6 mm) under remote shear loading (σ^o^_x__y_, σ^o^_x__z_ and σ^o^_yz_).

Two different orthotropic inclusions (Ort_06 and Ort_07) in Table 4 were used in the numerical calculation. It should be noted that the VIEM results represent average values of the normalized stresses in all the nodes of the VIEM model in Figure 16 and Figure 17. It should also be noted that the normalized shear stresses (σ_x__y_/σ^o^_x__y_, σ_x__z_/σ^o^_x__z_ and σ_yz_/σ^o^_yz_) inside the orthotropic oblate spheroidal inclusions were found to be constant, respectively [1]. Table 23 shows numerical solutions by the volume integral equation method for the normalized shear stresses (σ_x__y_/σ^o^_x__y_, σ_x__z_/σ^o^_x__z_ and σ_yz_/σ^o^_yz_) inside the orthotropic oblate spheroidal inclusions. For the inclusion in Ort_06, the normalized shear stresses (σ_x__y_/σ^o^_x__y_, σ_x__z_/σ^o^_x__z_ and σ_yz_/σ^o^_yz_) inside the inclusion were greater than 1.0, respectively. However, for the inclusion in Ort_07, the normalized shear stresses (σ_x__y_/σ^o^_x__y_, σ_x__z_/σ^o^_x__z_ and σ_yz_/σ^o^_yz_) inside the inclusion were less than 1.0, respectively. Figure 31 shows numerical solutions by the volume integral equation method for the normalized shear stresses (a) σ_x__y_/σ^o^_x__y_, (b) σ_x__z_/σ^o^_x__z_ and (c) σ_yz_/σ^o^_yz_ along (i) the x–axis inside (−6 mm ≤ x ≤ 6 mm) and (ii) the circumferential direction (0° ≤ θ (see Figure 16) ≤ 360°) of the orthotropic oblate spheroidal inclusions (b/a = c/a = 0.5 where a = 6 mm) under remote shear loading, σ^o^_x__y_, σ^o^_x__z_ and σ^o^_yz_. Figure 32 shows numerical solutions by the volume integral equation method for the normalized shear stresses (a) σ_x__y_/σ^o^_x__y_, (b) σ_x__z_/σ^o^_x__z_ and (c) σ_yz_/σ^o^_yz_ along (i) the x–axis inside (−6 mm ≤ x ≤ 6 mm) and (ii) the circumferential direction (0° ≤ θ (see Figure 17) ≤ 360°) of the orthotropic oblate spheroidal inclusions (b/a = c/a = 0.75 where a = 6 mm) under remote shear loading, σ^o^_x__y_, σ^o^_x__z_ and σ^o^_yz_. It was determined in Figure 31 and Figure 32 that the normalized shear stresses (σ_x__y_/σ^o^_x__y_, σ_x__z_/σ^o^_x__z_ and σ_yz_/σ^o^_yz_) inside the orthotropic oblate spheroidal inclusions are constant in all directions considered. Furthermore, even though, as shown in Figure 26, the cross-section in the xy plane is identical to the cross-section in the xz plane in the oblate spheroidal inclusion, since c_55_ (shear modulus in the xz plane) is greater than c_66_ (shear modulus in the xy plane) and c_66_ is greater than c_44_ (shear modulus in the yz plane) in the orthotropic inclusions of the Ort_06 and Ort_07 materials, the normalized shear stress, σ_x__y_/σ^o^_x__y_, was different from the normalized shear stress, σ_xz_/σ^o^_xz_, inside the orthotropic oblate spheroidal inclusion under remote shear loading.

From Figure 22, Figure 23, Figure 24, Figure 25, Figure 27, Figure 28, Figure 29, Figure 30, Figure 31 and Figure 32 and Table 17, Table 18, Table 19, Table 20, Table 21 and Table 22, it was determined that if the inclusion is harder than the matrix, the normalized shear stresses (σ_x__y_/σ^o^_x__y_, σ_x__z_/σ^o^_x__z_ and σ_yz_/σ^o^_yz_) inside the inclusion are greater than 1.0, respectively. It was also determined that if the inclusion is softer than the matrix, the normalized shear stresses (σ_x__y_/σ^o^_x__y_, σ_x__z_/σ^o^_x__z_ and σ_yz_/σ^o^_yz_) inside the inclusion are less than 1.0, respectively. 

From Figure 26, notable similarities are observed for isotropic inclusions. First, the cross-section in the xy plane of the isotropic prolate spheroidal inclusion is identical to the cross-section in the yz plane and is symmetrical to the cross-sections in the xy and xz planes of the isotropic oblate spheroidal inclusion. Second, the normalized shear stress, σ_x__y_/σ^o^_x__y_, inside the isotropic prolate spheroidal inclusion is identical to both the normalized shear stress, σ_yz_/σ^o^_yz_, inside the isotropic prolate spheroidal inclusion and the normalized shear stresses, σ_x__y_/σ^o^_x__y_ and σ_xz_/σ^o^_xz_, inside the isotropic oblate spheroidal inclusion under remote shear loading. Third, the cross-section in the xz plane of the isotropic prolate spheroidal inclusion is symmetrical to the cross-section in the yz plane of the isotropic oblate spheroidal inclusion. Fourth, the normalized shear stress, σ_x__z_/σ^o^_x__z_, inside the isotropic prolate spheroidal inclusion is identical to the normalized shear stress, σ_yz_/σ^o^_yz_, inside the isotropic oblate spheroidal inclusion under remote shear loading.

In contrast, certain differences can be seen for orthotropic inclusions. First, although the cross-section in the xy plane of the orthotropic prolate spheroidal inclusion is still symmetrical to the cross-section in the xy plane of the orthotropic oblate spheroidal inclusion, it is no longer identical to the cross-section in the yz plane of the orthotropic prolate spheroidal inclusion. Second, since the cross-section in the xy plane of the orthotropic prolate spheroidal inclusion is no longer symmetrical to the cross-section in the xz plane of the orthotropic oblate spheroidal inclusion, the normalized shear stress, σ_xy_/σ^o^_xy_, inside the orthotropic prolate spheroidal inclusion is only identical to the normalized shear stress, σ_xy_/σ^o^_xy_, inside the orthotropic oblate spheroidal inclusion under remote shear loading. Third, since the cross-section in the xz plane of the orthotropic prolate spheroidal inclusion is no longer symmetrical to the cross-section in the yz plane of the orthotropic oblate spheroidal inclusion, the normalized shear stress, σ_xz_/σ^o^_xz_, inside the orthotropic prolate spheroidal inclusion is not identical to the normalized shear stress, σ_yz_/σ^o^_yz_, inside the orthotropic oblate spheroidal inclusion under remote shear loading.

It should be noted that, through numerical analysis using the volume integral equation method, we could quantitatively verify two qualitative predictions: (1) the normalized shear stresses (σ_xy_/σ^o^_xy_, σ_xz_/σ^o^_xz_ and σ_yz_/σ^o^_yz_) inside the orthotropic spherical inclusions are different from each other, and (2) for orthotropic spheroidal inclusions, there exists only one symmetrical cross-section when the remote loadings are shear (σ^o^_xy_, σ^o^_xz_ and σ^o^_yz_).

It was determined that values of the normalized tensile stress (σ_xx_/σ^o^_xx_) or the normalized shear stresses (σ_xy_/σ^o^_xy_, σ_xz_/σ^o^_xz_ and σ_yz_/σ^o^_yz_) inside the isotropic spheroidal inclusions differed significantly from those inside the orthotropic spheroidal inclusions. Therefore, thorough investigation of spheroidal inclusion problems requires stress analysis for both anisotropic spheroidal inclusion problems and isotropic spheroidal inclusion problems.

We also considered multiple isotropic/anisotropic spheroidal inclusions in an infinite isotropic matrix subject to uniform remote tensile loading, σ^o^_xx_. In a future paper, the authors will introduce the VIEM solutions of multiple isotropic/orthotropic spheroidal inclusions in an infinite isotropic matrix under arbitrary loading conditions. It is obvious that general characteristics of multiple isotropic/anisotropic inclusion problems cannot be fully analyzed from the basic characteristics of the corresponding single or two isotropic/anisotropic inclusion problems. Therefore, applying multiple inclusion problems to a wide class of real composite materials and structures requires extending the analysis to multiple isotropic/anisotropic inclusions of different shapes.

Both the standard finite element method (FEM) and the boundary element method (BEM) are powerful general-purpose tools in the field of numerical analysis. Since the VIEM is a combination of these two methods, it is also highly beneficial to the field of numerical analysis and can play a very important role in solving “multiple inclusion problems”. The authors hope that the results using the VIEM cited in this paper will be used as benchmarked data for verifying the results of similar research using other analytical and numerical methods.

## 4. Conclusions

In order to introduce the VIEM as a versatile numerical method for the three-dimensional elastostatic inclusion problem, it was applied to a class of three-dimensional elastostatic inclusion problems. We first considered single isotropic/orthotropic spherical, prolate (with an aspect ratio of 0.5 and 0.75) and oblate (with an aspect ratio of 0.5 and 0.75) spheroidal inclusions in an infinite isotropic matrix under uniform remote tensile loading. Thirteen inclusions with different characteristics were considered in the numerical calculation. Excellent agreement was found between the analytical and numerical solutions using the VIEM for single isotropic spherical inclusion problems. It was determined that the normalized tensile stress (σ_xx_/σ^o^_xx_) inside the isotropic/orthotropic spherical, prolate and oblate spheroidal inclusions was constant in two different directions (x–axis and circumferential direction). When the inclusion is harder than the matrix, the normalized tensile stress (σ_xx_/σ^o^_xx_) inside the inclusion can be arranged in ascending order of magnitude: (1) prolate spheroidal inclusion (a/b = c/b = 0.5), (2) prolate spheroidal inclusion (a/b = c/b = 0.75), (3) sphere, (4) oblate spheroidal inclusion (b/a = c/a = 0.75) and (5) oblate spheroidal inclusion (b/a = c/a = 0.5).

We next considered single isotropic/orthotropic spherical, prolate (with an aspect ratio of 0.5 and 0.75) and oblate (with an aspect ratio of 0.5 and 0.75) spheroidal inclusions in an infinite isotropic matrix under remote shear loading. Five inclusions with different characteristics were considered in the numerical calculation. It was determined that the normalized shear stresses (σ_x__y_/σ^o^_x__y_, σ_x__z_/σ^o^_x__z_ and σ_yz_/σ^o^_yz_) inside the isotropic/orthotropic spherical, prolate and oblate spheroidal inclusions were constant in two different directions (x–axis and circumferential direction), respectively. When the inclusion was harder than the matrix, the normalized shear stresses (σ_xy_/σ^o^_xy_, σ_xz_/σ^o^_xz_ and σ_yz_/σ^o^_yz_) inside the inclusion were greater than 1.0, respectively. Furthermore, for isotropic spheroidal inclusions, there existed two identical or symmetrical cross-sections, while for orthotropic spheroidal inclusions, there existed only one symmetric cross-section when the remote loadings were shear (σ^o^_xy_, σ^o^_xz_ and σ^o^_yz_).

It is the authors’ hope that the present solutions for various types of inclusions with different material properties under different loading conditions using the parallel volume integral equation method will be established as reference values for verifying the results of other analytical and numerical methods.

It was also determined that applying multiple inclusion problems to a wide class of real composite materials and structures requires extending the analysis to multiple isotropic/anisotropic inclusions of different numbers and shapes. The parallel volume integral equation method (PVIEM) is now generally more applicable and executable than the standard finite element or boundary element methods. Subsequently, the PVIEM can be used to calculate other quantities of practical interest in realistic models of composites containing isotropic or anisotropic inclusions of arbitrary shapes under arbitrary loading conditions.

It should also be pointed out that, since the VIEM is a combination of the FEM and the BEM, it may have an unknown advantage that neither the FEM model nor the BEM model alone possess. For example, although certain VIEM models are incorrect from the point of view of the standard FEM only, they can be correctly implemented in the VIEM. In a future paper, the authors will attempt to provide more distinct examples to support this new finding. Finally, as a new machine learning-based predictive framework has been proposed for the accurate and efficient evaluation of singular integrals in the boundary element method (BEM) [32], of particular interest to researchers going forward will be the development of a general-purpose machine learning framework for predicting singular integrals [29] in the volume integral equation method.

## Figures and Tables

**Table 1:** Capabilities of VIEMAP.

	Two Dimensional		Three Dimensional	
ViemMesh (Pre-Processor)	(1) 8-node quadrilateral finite element (2) 6-node triangular finite element		(1) 20-node hexahedral finite element (2) 10-node tetrahedral finite element	
VIEM (Solver)	**Multiple Inclusion Problems**		**Multiple Inclusion Problems**	
	**Isotropic Inclusions**	**Anisotropic Inclusions**	**Isotropic Inclusions**	**Anisotropic Inclusions**
	(1) Elastostatic solver (2) Elastodynamic solver		(1) Elastostatic solver (2) Elastodynamic solver	
ViemPlot (Post-Processor)	(1) Displacement contour plot (2) Stress contour plot		(1) Displacement contour plot (2) Stress contour plot	

**Table 2:** Material Properties of the Isotropic Matrix and the Isotropic Inclusions.

Material	λ (GPa)	μ (GPa)	E (GPa)	ν
Matrix (Iso_01)	67.3401	37.8788	100.0	0.32
Inclusion (Iso_01)	176.060	176.060	440.15	0.25
Matrix (Iso_02)	121.154	80.7692	210.0	0.30
Inclusion (Iso_02)	83.1643	176.724	410.0	0.16
Matrix (Iso_03)	75.0	37.5	100.0	0.3333
Inclusion (Iso_03)	150.0	75.0	200.0	0.3333
Matrix (Iso_04)	75.0	37.5	100.0	0.3333
Inclusion (Iso_04)	375.0	187.5	500.0	0.3333
Matrix (Iso_05)	75.0	37.5	100.0	0.3333
Inclusion (Iso_05)	750.0	375.0	1000.0	0.3333
Matrix (Iso_06)	121.154	80.7692	210.0	0.30
Inclusion (Iso_06)	87.2202	41.0448	110.0	0.34
Matrix (Iso_07)	75.0	37.5	100.0	0.3333
Inclusion (Iso_07)	15.0	7.5	20.0	0.3333
Matrix (Iso_08)	75.0	37.5	100.0	0.3333
Inclusion (Iso_08)	52.5	26.25	70.0	0.3333

**Table 3:** Material Properties of the Isotropic Matrix and the Orthotropic Inclusions.

Unit: GPa	Orthotropic Inclusions					Isotropic Matrix
	Ort_01	Ort_02	Ort_03	Ort_04	Ort_05	
c_11_	139.54	279.08	418.61	41.86	69.77	143.10
c_12_ = c_21_	3.90	7.80	11.7	1.17	1.95	67.34
c_13_ = c_31_	3.90	7.80	11.7	1.17	1.95	67.34
c_22_	15.28	30.56	45.83	4.58	7.64	143.10
c_23_ = c_32_	3.29	6.59	9.88	0.99	1.65	67.34
c_33_	15.28	30.56	45.83	4.58	7.64	143.10
c_44_	5.90	11.80	17.70	1.77	2.95	37.88
c_55_	5.90	11.80	17.70	1.77	2.95	37.88
c_66_	5.90	11.80	17.70	1.77	2.95	37.88

**Table 4:** Material properties of the isotropic matrix and the orthotropic inclusions.

Unit: GPa	Orthotropic Inclusions		Isotropic Matrix
	Ort_06	Ort_07	
c_11_	61.11	458.30	143.10
c_12_ = c_21_	17.95	134.63	67.34
c_13_ = c_31_	20.54	154.02	67.34
c_22_	32.77	245.78	143.10
c_23_ = c_32_	15.05	112.87	67.34
c_33_	47.89	359.15	143.10
c_44_	9.97	74.79	37.88
c_55_	15.16	113.69	37.88
c_66_	10.99	82.40	37.88

**Table 5:** Material Property Characteristics.

Material		Characteristics	
Matrix (Iso_01)	Isotropic	No restriction in Poisson’s ratio	E(Inclusion) > E(Matrix)
Inclusion (Iso_01)	Isotropic	No restriction in Poisson’s ratio	
Matrix (Iso_02)	Isotropic	No restriction in Poisson’s ratio	E(Inclusion) > E(Matrix)
Inclusion (Iso_02)	Isotropic	No restriction in Poisson’s ratio	
Matrix (Iso_03)	Isotropic	ν = 1/3	E(Inclusion) > E(Matrix)
Inclusion (Iso_03)	Isotropic	ν = 1/3	
Matrix (Iso_04)	Isotropic	ν = 1/3	E(Inclusion) > E(Matrix)
Inclusion (Iso_04)	Isotropic	ν = 1/3; E(Iso_04) > E(Iso_03)	
Matrix (Iso_05)	Isotropic	ν = 1/3	E(Inclusion) > E(Matrix)
Inclusion (Iso_05)	Isotropic	ν = 1/3; E(Iso_05) > E(Iso_04)	
Matrix (Iso_06)	Isotropic	No restriction in Poisson’s ratio	E(Inclusion) < E(Matrix)
Inclusion (Iso_06)	Isotropic	No restriction in Poisson’s ratio	
Matrix (Iso_07)	Isotropic	ν = 1/3	E(Inclusion) < E(Matrix)
Inclusion (Iso_07)	Isotropic	ν = 1/3	
Matrix (Iso_08)	Isotropic	ν = 1/3	E(Inclusion) < E(Matrix)
Inclusion (Iso_08)	Isotropic	ν = 1/3; E(Iso_08) > E(Iso_07)	
Matrix (Ort_01)	Isotropic	No restriction in Poisson’s ratio	
Inclusion (Ort_01)	Orthotropic	c_11_ > c_22_ = c_33_	
Matrix (Ort_02)	Isotropic	No restriction in Poisson’s ratio	
Inclusion (Ort_02)	Orthotropic	c_11_ > c_22_ = c_33_; c_11_(Ort_02) > c_11_(Ort_01)	
Matrix (Ort_03)	Isotropic	No restriction in Poisson’s ratio	
Inclusion (Ort_03)	Orthotropic	c_11_ > c_22_ = c_33_; c_11_(Ort_03) > c_11_(Ort_02)	
Matrix (Ort_04)	Isotropic	No restriction in Poisson’s ratio	
Inclusion (Ort_04)	Orthotropic	c_11_ > c_22_ = c_33_; c_11_(Ort_04) < c_11_(Ort_01)	
Matrix (Ort_05)	Isotropic	No restriction in Poisson’s ratio	
Inclusion (Ort_05)	Orthotropic	c_11_ > c_22_ = c_33_; c_11_(Ort_04) < c_11_(Ort_05) < c_11_(Ort_01)	
Matrix (Ort_06)	Isotropic	No restriction in Poisson’s ratio	
Inclusion (Ort_06)	Orthotropic	μ (Matrix) > c_55_ (Inclusion) > c_66_ (Inclusion) > c_44_ (Inclusion)	
Matrix (Ort_07)	Isotropic	No restriction in Poisson’s ratio	
Inclusion (Ort_07)	Orthotropic	c_55_ (Inclusion) > c_66_ (Inclusion) > c_44_ (Inclusion) > μ (Matrix)	

**Table 6:** Normalized tensile stress component (σ_xx_/σ^o^_xx_) within the isotropic spherical inclusion due to uniform remote tensile loading (σ^o^_xx_).

Material	VIEM (Average)	Analytical Solution	Error (%)
Iso_01	1.5800	-	-
Iso_02	1.2823	1.2822	0.0078

**Table 7:** Normalized tensile stress component (σ_xx_/σ^o^_xx_) within the isotropic spherical inclusion due to uniform remote tensile loading (σ^o^_xx_).

Material	VIEM (Average)	Analytical Solution	Error (%)
Iso_03	1.3090	1.3091	0.0076
Iso_04	1.6171	1.6173	0.0124
Iso_05	1.7582	1.7582	0.0

**Table 8:** Normalized tensile stress component (σ_xx_/σ^o^_xx_) within the isotropic spherical inclusion due to uniform remote tensile loading (σ^o^_xx_).

Material	VIEM (Average)	Analytical Solution	Error (%)
Iso_06	0.7200	0.7200	0.0
Iso_07	0.3557	0.3556	0.0281
Iso_08	0.8343	0.8343	0.0

**Table 9:** Normalized tensile stress component (σ_xx_/σ^o^_xx_) within the orthotropic spherical inclusion due to uniform remote tensile loading (σ^o^_xx_).

Material	VIEM (Average)
Ort_01	1.1520
Ort_02	1.4536
Ort_03	1.5910
Ort_04	0.5836
Ort_05	0.8129

**Table 10:** Normalized tensile stress component (σ_xx_/σ^o^_xx_) within the isotropic prolate spheroidal inclusion due to uniform remote tensile loading (σ^o^_xx_).

Material	VIEM (Average)	
	a/b = c/b = 0.5 (see Figure 5)	a/b = c/b = 0.75 (see Figure 5)
Iso_01	1.4268	1.5028
Iso_02	1.2177	1.2500

**Table 11:** Normalized tensile stress component (σ_xx_/σ^o^_xx_) within the isotropic prolate spheroidal inclusion due to uniform remote tensile loading (σ^o^_xx_).

Material	VIEM (Average)	
	a/b = c/b = 0.5 (See Figure 5)	a/b = c/b = 0.75 (See Figure 5)
Iso_03	1.2374	1.2736
Iso_04	1.4502	1.5330
Iso_05	1.5409	1.6477

**Table 12:** Normalized tensile stress component (σ_xx_/σ^o^_xx_) within the isotropic prolate spheroidal inclusion due to uniform remote tensile loading (σ^o^_xx_).

	VIEM (Average)	
	a/b = c/b = 0.5 (See Figure 5)	a/b = c/b = 0.75 (See Figure 5)
Iso_06	0.7613	0.7397
Iso_07	0.4042	0.3780
Iso_08	0.8610	0.8471

**Table 13:** Normalized Tensile Stress Component (σ_xx_/σ^o^_xx_) within the Orthotropic Prolate Spheroidal Inclusion due to Uniform Remote Tensile Loading (σ^o^_xx_).

Material	VIEM (Average)	
	a/b = c/b = 0.5 (See Figure 5)	a/b = c/b = 0.75 (See Figure 5)
Ort_01	1.1244	1.1385
Ort_02	1.3546	1.4038
Ort_03	1.4519	1.5202
Ort_04	0.6246	0.6027
Ort_05	0.8375	0.8246

**Table 14:** Normalized tensile stress component (σ_xx_/σ^o^_xx_) within the isotropic oblate spheroidal inclusion due to uniform remote tensile loading (σ^o^_xx_).

Material	VIEM (Average)	
	b/a = c/a = 0.5 (See Figure 5)	b/a = c/a = 0.75 (See Figure 5)
Iso_01	2.1363	1.7790
Iso_02	1.4811	1.3599

**Table 15:** Normalized tensile stress component (σ_xx_/σ^o^_xx_) within the isotropic oblate spheroidal inclusion due to uniform remote tensile loading (σ^o^_xx_).

Material	VIEM (Average)	
	b/a = c/a = 0.5 (See Figure 5)	b/a = c/a = 0.75 (See Figure 5)
Iso_03	1.5251	1.3938
Iso_04	2.2350	1.8413
Iso_05	2.6483	2.0556

**Table 16:** Normalized tensile stress component (σ_xx_/σ^o^_xx_) within the isotropic oblate spheroidal inclusion due to uniform remote tensile loading (σ^o^_xx_).

Material	VIEM (Average)	
	b/a = c/a = 0.5 (See Figure 5)	b/a = c/a = 0.75 (See Figure 5)
Iso_06	0.6310	0.6793
Iso_07	0.2695	0.3134
Iso_08	0.7733	0.8072

**Table 17:** Normalized tensile stress component (σ_xx_/σ^o^_xx_) within the orthotropic oblate spheroidal inclusion due to uniform remote tensile loading (σ^o^_xx_).

Material	VIEM (Average)	
	b/a = c/a = 0.5 (See Figure 5)	b/a = c/a = 0.75 (See Figure 5)
Ort_01	1.2292	1.1833
Ort_02	1.7864	1.5780
Ort_03	2.1040	1.7745
Ort_04	0.5006	0.5453
Ort_05	0.7570	0.7882

**Table 18:** Normalized shear stress components (σ_x__y_/σ^o^_x__y_, σ_x__z_/σ^o^_x__z_ and σ_yz_/σ^o^_yz_) within the isotropic spherical inclusion due to remote shear loading (σ^o^_x__y_, σ^o^_x__z_ and σ^o^_yz_).

Material	VIEM (Average)		
	σ_x__y_/σ^o^_x__y_	σ_xz_/σ^o^_xz_	σ_yz_/σ^o^_yz_
Iso_01	1.7109	1.7109	1.7109
Iso_05	1.9231	1.9231	1.9231
Iso_06	0.6636	0.6636	0.6636

**Table 19:** Normalized shear stress components (σ_x__y_/σ^o^_x__y_, σ_x__z_/σ^o^_x__z_ and σ_yz_/σ^o^_yz_) within the orthotropic spherical inclusion due to remote shear loading (σ^o^_x__y_, σ^o^_x__z_ and σ^o^_yz_).

Material	VIEM (Average)		
	σ_x__y_/σ^o^_x__y_	σ_xz_/σ^o^_xz_	σ_yz_/σ^o^_yz_
Ort_06	1.4006	1.5456	1.3537
Ort_07	0.4356	0.5576	0.4030

**Table 20:** Normalized shear stress components (σ_x__y_/σ^o^_x__y_, σ_x__z_/σ^o^_x__z_ and σ_yz_/σ^o^_yz_) within the isotropic prolate spheroidal inclusion due remote shear loading (σ^o^_x__y_, σ^o^_x__z_ and σ^o^_yz_).

Material	VIEM (Average)					
	a/b = c/b = 0.5 (See Figure 5)			a/b = c/b = 0.75 (See Figure 5)		
	σ_xy_/σ^o^_xy_	σ_xz_/σ^o^_xz_	σ_yz_/σ^o^_yz_	σ_xy_/σ^o^_xy_	σ_xz_/σ^o^_xz_	σ_yz_/σ^o^_yz_
Iso_01	1.7619	1.5329	1.7619	1.7490	1.6214	1.7490
Iso_05	1.9935	1.6765	1.9935	1.9772	1.7972	1.9772
Iso_06	0.6538	0.7036	0.6538	0.6565	0.6820	0.6565

**Table 21:** Normalized shear stress components (σ_x__y_/σ^o^_x__y_, σ_x__z_/σ^o^_x__z_ and σ_yz_/σ^o^_yz_) within the orthotropic prolate spheroidal inclusion due remote shear loading (σ^o^_x__y_, σ^o^_x__z_ and σ^o^_yz_).

Material	VIEM (Average)					
	a/b = c/b = 0.5 (See Figure 5)			a/b = c/b = 0.75 (See Figure 5)		
	σ_xy_/σ^o^_xy_	σ_xz_/σ^o^_xz_	σ_yz_/σ^o^_yz_	σ_xy_/σ^o^_xy_	σ_xz_/σ^o^_xz_	σ_yz_/σ^o^_yz_
Ort_06	1.4239	1.4192	1.3735	1.4180	1.4828	1.3685
Ort_07	0.4258	0.6010	0.3934	0.4282	0.5774	0.3957

**Table 22:** Normalized shear stress components (σ_x__y_/σ^o^_x__y_, σ_x__z_/σ^o^_x__z_ and σ_yz_/σ^o^_yz_) within the isotropic oblate spheroidal inclusion due remote shear loading (σ^o^_x__y_, σ^o^_x__z_ and σ^o^_yz_).

Material	VIEM (Average)					
	b/a = c/a = 0.5 (See Figure 5)			b/a = c/a = 0.75 (See Figure 5)		
	σ_xy_/σ^o^_xy_	σ_xz_/σ^o^_xz_	σ_yz_/σ^o^_yz_	σ_xy_/σ^o^_xy_	σ_xz_/σ^o^_xz_	σ_yz_/σ^o^_yz_
Iso_01	1.7619	1.7619	1.5329	1.7490	1.7490	1.6214
Iso_05	1.9935	1.9935	1.6765	1.9772	1.9772	1.7972
Iso_06	0.6538	0.6538	0.7036	0.6565	0.6565	0.6820

**Table 23:** Normalized shear stress components (σ_x__y_/σ^o^_x__y_, σ_x__z_/σ^o^_x__z_ and σ_yz_/σ^o^_yz_) within the orthotropic oblate spheroidal inclusion due remote shear loading (σ^o^_x__y_, σ^o^_x__z_ and σ^o^_yz_).

Material	VIEM (Average)					
	b/a = c/a = 0.5 (See Figure 5)			b/a = c/a = 0.75 (See Figure 5)		
	σ_xy_/σ^o^_xy_	σ_xz_/σ^o^_xz_	σ_yz_/σ^o^_yz_	σ_xy_/σ^o^_xy_	σ_xz_/σ^o^_xz_	σ_yz_/σ^o^_yz_
Ort_06	1.4239	1.5808	1.2798	1.4180	1.5719	1.3175
Ort_07	0.4258	0.5477	0.4465	0.4282	0.5501	0.4226

## Data Availability

The data presented in this study are available upon request from the corresponding author.
